# Formal nomenclature and description of cryptic species of the *Encyrtus sasakii* complex (Hymenoptera: Encyrtidae)

**DOI:** 10.1038/srep34372

**Published:** 2016-10-04

**Authors:** Ying Wang, Qing-Song Zhou, Hui-Jie Qiao, Ai-Bing Zhang, Fang Yu, Xu-Bo Wang, Chao-Dong Zhu, Yan-Zhou Zhang

**Affiliations:** 1 Key Laboratory of Zoological Systematics and Evolution, Institute of Zoology, Chinese Academy of Sciences, Beijing 100101, China; 2 University of Chinese Academy of Sciences (UCAS), No. 19A Yuquan Road, Beijing 100049, China; 3 College of Life Sciences, Capital Normal University, Beijing 100048, China; 4 Key Laboratory for Silviculture and Conservation of Ministry of Education, Beijing Forestry University, Beijing 100083, China

## Abstract

With the recent development of molecular approaches to species delimitation, a growing number of cryptic species have been discovered in what had previously been thought to be single morpho-species. Molecular methods, such as DNA barcoding, have greatly enhanced our knowledge of taxonomy, but taxonomy remains incomplete and needs a formal species nomenclature and description to facilitate its use in other scientific fields. A previous study using DNA barcoding, geometric morphometrics and mating tests revealed at least two cryptic species in the *Encyrtus sasakii* complex. (Hymenoptera: Encyrtidae). To describe these two new species formally (*Encyrtus eulecaniumiae* sp. nov. and *Encyrtus rhodococcusiae* sp. nov.), a detailed morphometric study of *Encyrtus* spp. was performed in addition to the molecular analysis and evaluation of biological data. Morphometric analyses, a multivariate ratio analysis (MRA) and a geometric morphometric analysis (GMA) revealed a great number of differences between the species, but reliable characteristics were not observed for diagnosing the cryptic species. We thus diagnosed these three *Encyrtus* species on the basis of the characteristics that resulted from genetic markers (mitochondrial cytochrome c oxidase subunit I and nuclear 28S rRNA) and biological data. A formal nomenclature and description of cryptic species was provided on the basis of an integrated taxonomy.

Over the past decade, DNA barcoding^1^ has been broadly used for the species identification of metazoans^2^,^3^,^4^. Unexpectedly, a large number of cryptic species were discovered for those species, which were considered to be a single morpho-species, particularly in the morphologically conserved invertebrate taxa, such as Lepidoptera^5^,^6^, Coleoptera^7^, Diptera^8^, and Hymenoptera^9^,^10^. As far as we know, almost all of these cryptic species were characterized by DNA barcodes. Without a formal nomenclature and description, these “identified species” cannot linked to the Linnaean System and associated with the established biological knowledge^11^,^12^,^13^,^14^,^15^; thus, the potential value of these barcodes to various research fields^16^,^17^,^18^ might be limited. Diagnostic characteristics could be provided by the DNA barcodes^15^,^19^ but only after scientific names have been given to the barcoded specimens (using morphology or any other means that had previously been used to diagnose the species). After these determinations have been made, diagnosis can continue using barcodes. Moreover, a stable nomenclature at the level of formal species can undoubtedly facilitate effective transfer of knowledge and again provide a foundation for more information associated with it.

Tentative strategies have been proposed and developed to resolve cryptic species and describe them^20^,^21^. Brower^22^ used only DNA barcodes (i.e., the mitochondrial cytochrome oxidase subunit I [*COI*] gene) for diagnosis and offered no formal morphological description. Recent attempts to integrate DNA and morphological taxonomy to delimitate species have provided a much better approach for cryptic species description^23^,^24^,^25^,^26^. An increasing number of authors have realized that during the description of cryptic species, morphological characteristics should be rigorously investigated and included in addition to DNA and biological data^27^,^28^. Due to similar external anatomical characteristics and continuous and/or overlapping morphological characteristics^24^,^29^,^30^,^31^,^32^,^33^, thorough morphological analyses, such as multivariate ratio analysis (MRA)^34^ and Geometric Morphometric Analysis (GMA), should be performed to demonstrate the variation among these cryptic species.

*Encyrtus* is a species-rich genus of Encyrtidae (Hymenoptera: Chalcidoidea) that currently contains approximately 90 described species^35^. Although many studies have contributed to the identification of these parasitoids, reliable distinctions and identifications are difficult to make because of their small size and similar external morphology, particularly for cryptic species or those in species complexes. In a previous study, a combined analysis of the geometric morphometrics of insect forewings, DNA data and mating tests were used to detect the existence of two cryptic species in the *Encyrtus sasakii* species complex in China^36^. For a complete taxonomy of these cryptic species, we obtained samples from six more populations in China (Supplementary Table S1) in addition to the samples described in Chesters *et al*.^36^. In this study, we further explored the patterns of morphological variation (e.g., the antenna characteristics and forewing vein dimensions) along with the genetic variation and biological data. Corroboration from independent data, including morphology, molecules and biology, provide a basis for the delimitation and formal nomenclature of these cryptic species.

## Results

Approximately 4,000 samples of the *E. sasakii* species complex were reared from four scale insect species, known as *Eulecanium kuwanai* (Kuwana), *Eulecanium giganteum* (Shinji), *Takahashia japonica* (Cockerell) and *Rhodococcus sariuoni* Borchsenius. The 167 female specimens were randomly selected from different populations according to their population size (details in Supplementary Table S1). Of these specimens, 135 were used in the Morphometric Analysis and MRA, 125 were used in the GMA (Geometric Morphometric Analysis) and 128 were used in the molecular analyses. Based on the results of the current work and on previous studies^36^, the specimens that emerged from *Takahashia japonica* were identified as *Encyrtus sasakii*, those from *Eulecanium kuwanai* and *E. giganteum* were identified as *Encyrtus eulecaniumiae* sp. nov., and those from *Rhodococcus sariuoni* were identified as *Encyrtus rhodococcusiae* sp. nov.

### Morphometric analysis

**Figure 1:**
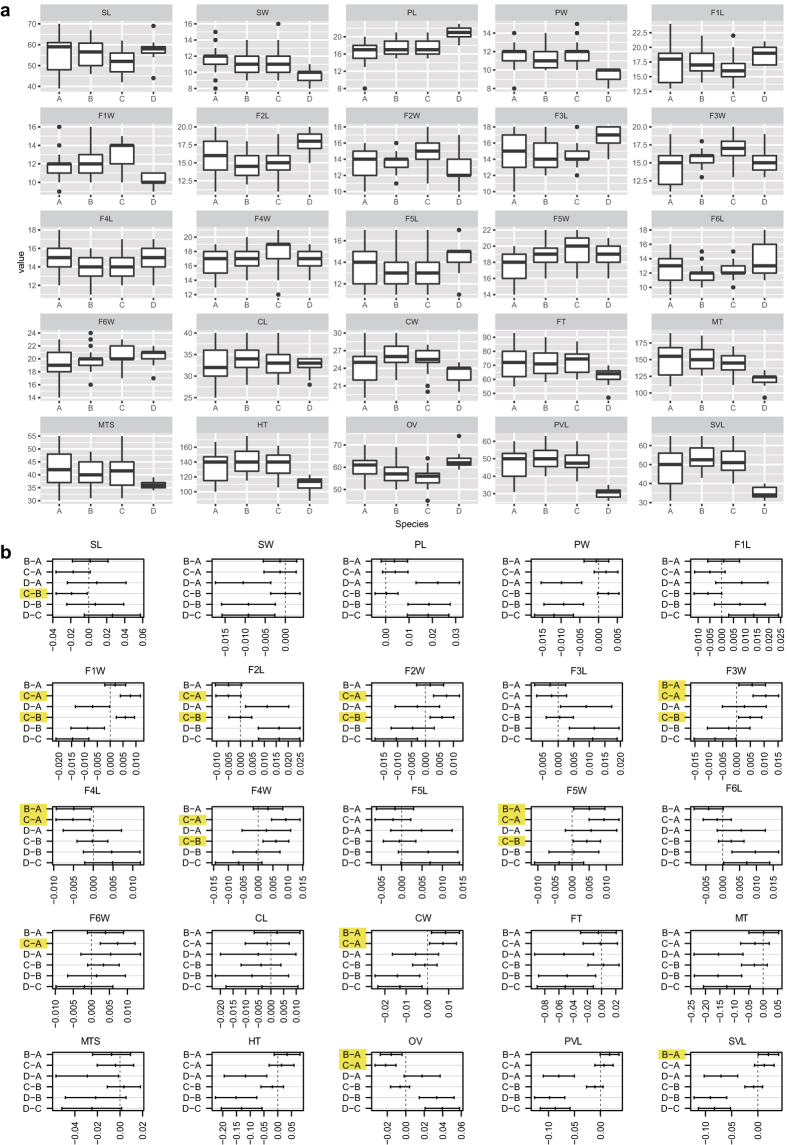
(**a**) Boxplot of the distribution of the measurements of 25 characters (for Abbreviations of the Character names, see Table 1). The horizontal thick bar represents the median value, the box includes 50% of the data, the whiskers extend to cover 95% of the distribution. (**b**) Post hoc comparisons of each morphological character using Tukey HSD test at 95% family-wise confidence level (no significant differences if zero included in confidence intervals), significant differences between characteristics of A, B and C are highlighted with yellow. A = *E. eulecaniumiae*, B = *E. rhodococcusiae*, C = *E. sasakii*, and D = *E. infelix*.

The boxplots (Fig. 1a) show that all 25 of the morphological characteristics (Table 1) are continuous, and they vary from slightly to distinctly overlapping between *Encyrtus sasakii*, *E. eulecaniumiae* and *E. rhodococcusiae*. However, statistical analysis of the variance showed that some of the morphological characteristics of these species are significantly different (Fig. 1b). Six characteristic values (F3W, F4L, F5W, CW, OV and SVL) were significantly different (P < 0.05) between *E. eulecaniumiae* and *E. rhodococcusiae*. Ten characteristic values (F1W, F2L, F2W, F3W, F4L, F4W, F5W, F6W, CW and OV) were significantly different (P < 0.05) between *E. eulecaniumiae* and *E. sasakii*. Seven characteristic values (SL, F1W, F2L, F2W, F3W, F4W and F5W) were significantly different (P < 0.05) between *E. rhodococcusiae* and *E. sasakii*. Moreover, two characteristic values (F3W and F5W) were significantly different among all three species pairs.

### Multivariate ratio analysis (MRA)

**Figure 2:**
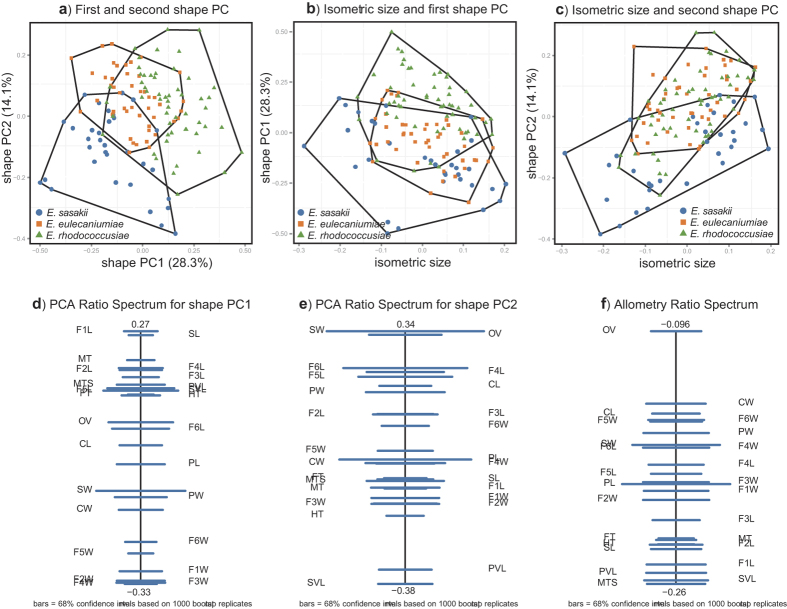
Results of multivariate ratio analysis (MRA) for females of *Encyrtus sasakii*, *E*. *eulecaniumiae* and *E*. *rhodococcusiae*. **a**) Scatterplot of a principal component analysis (PCA) in shape space. (**b**) Isometric size versus first principal component in shape space. (**c**) Isometric size versus second principal component in shape space. (**d**) PCA ratio spectrum of the first principal component. (**e**) PCA ratio spectrum of the second principal component. (**f**) The allometry ratio spectrum. Confidence intervals [horizontal bars in (**d,e,f**), were estimated with a bootstrap of the original values of z directly from the empirical distribution.

The variables with matrix scatterplots and Pearson product-moment correlation coefficients were checked, and no variables were found to correlate more or less strongly than others (Supplementary Table S2 and Figure S1). Thus, all variables are included in the subsequent analyses. The first eleven principal components (PC) included 84.9% of all informative variations (Supplementary Table S3). The scatterplot of the first two shape PCs (which included 42.4% of all informative variations) showed that the three species were partially overlapping along the forms of the chosen components (Fig. 2a). Figure 2b,c displays results similar to those of Fig. 2a, and this is also the case for the rest of the PCs. In the scatterplots of isosize against the first and second shape PC, *E. sasakii* was slightly smaller on average than *E. eulecaniumiae*, and the latter was slightly smaller on average than *E. rhodococcusiae*, although the size ranges overlapped broadly (Fig. 2b,c).

On the PCA (principal component analysis) ratio spectrum of the first shape PC (Fig. 2d), some of the variation was explained by ratios, such as F1L:F4W and SL:F4W, which corresponded to points located close to the opposite ends of the spectrum. The first PCA ratio spectrum was dominated by ratios relating to relative funicular segment lengths. The second PCA ratio (Fig. 2e) was dominated by the ratios SW/SVL, SW/PVL, OV/SVL, and OV/PVL, and these characteristics were related to the forewing veins (SVL, PVL), antennal scape (SW) and ovipositor (OV). The last two ratios were also among the most important ratios in the allometry ratio spectrum (Fig. 2f).

**Figure 3:**
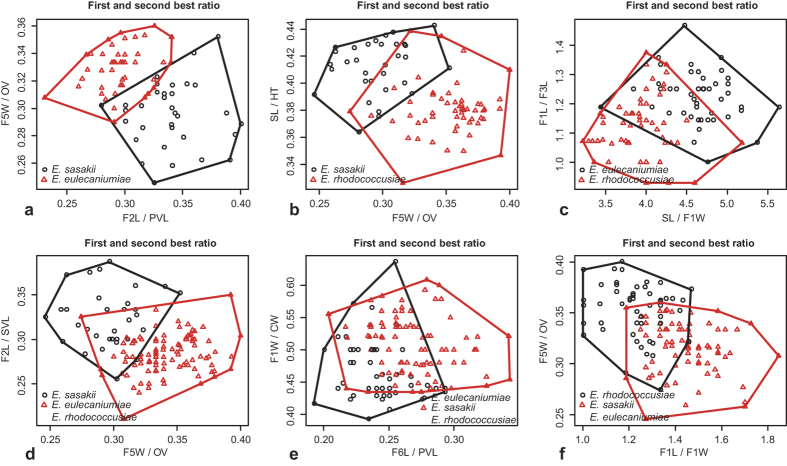
Scatterplots of the two most discriminating ratios for females of *Encyrtus sasakii*, *E*. *eulecaniumiae* and *E*. *rhodococcusiae*. Plots (**a–f**) show the first versus second ratio for each comparison of the three *Encyrtus* species.

The LDA (linear discriminant analysis) ratio extractor^37^ was used to determine the optimal body ratios to separate the species, and the results are compiled in Table 2. The ranges of first- and second-best ratios were mostly overlapping between the respective groupings. The F5W/OV ratio was assigned as the best ratio for discriminating among *E. sasakii*, *E. eulecaniumiae* and *E. rhodococcusiae*. For all the comparisons, the δ, which is a measure of how well the shape discriminates in comparison with the size^37^, was close to 0 (0.01–0.04), indicating that the separation of these three species was due mainly to shape rather than size. However, the ranges of the first- and second-best ratios often overlapped between the respective groupings (Fig. 3a–f), which indicates that those character ratios could not be used to represent important diagnostic features. The standard distances ranged from 2.62 to 4.90, which does not reflect a good separation of these three species, as observed in the shape PCA.

### Geometric morphometric analysis (GMA)

**Figure 4:**
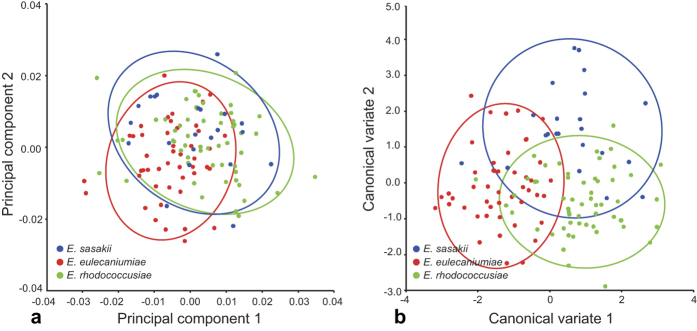
(**a**) Scatter plots constructed from principal component analyses (PCA) of the data set of seven landmarks from the forewings of the three *Encyrtus* species. In the scatter plots, the first and second principal components are plotted on the x and y axis, respectively. (**b**) Scatter plots constructed from canonical variate analysis (CVA) of the landmark data set.

A total of 125 specimens were prepared for GMA (Supplementary Table S1). The partial or fuzzy separation of the populations was observed for specimens on the first two principal axes (Fig. 4a,b). The first two components contributed to 47.32% of the total variance (PC1 = 25.50% and PC2 = 21.82%). The third, fourth and fifth components contributed to 18.20%, 10.60%, and 8.43% of the total variance, respectively, which did not improve the separation of the populations.

### Analysis of molecular data

In this study, we employed 120 sequences of the mitochondrial cytochrome oxidase subunit I (COI) gene and 76 sequences of the 28S ribosomal gene (including the 69 samples used in Chesters *et al*.^36^; Supplementary Table S1). After edge trimming, the data matrix consisted of 557 bp for COI and 503 bp for 28S. As mentioned in a previous study, the 28S sequences of these three provisional species have a minor divergence from one another. *E. rhodococcusiae* has a one-bp difference, with the other two at position 217 (“C” compared with “T”), and *E. eulecaniumiae* has a one-bp difference, with the other two at position 451 (“A” compared with “G”). Conversely, the COI degree of genetic divergence was particularly high. A barcode gap was discovered between the maximum intraspecific variation (K2P distance: 3.33%; p-distance: 3.23%) and the minimum interspecific divergence (K2P distance: 8.81%; p-distance: 8.26%) (Table 3). The application of character-based methods can provide a combination of diagnostic nucleotides that can be used to identify the three cryptic species correctly. The species-specific nucleotide positions of COI (pure diagnostic barcode characters) for the three cryptic species are listed in Table 4.

**Figure 5:**
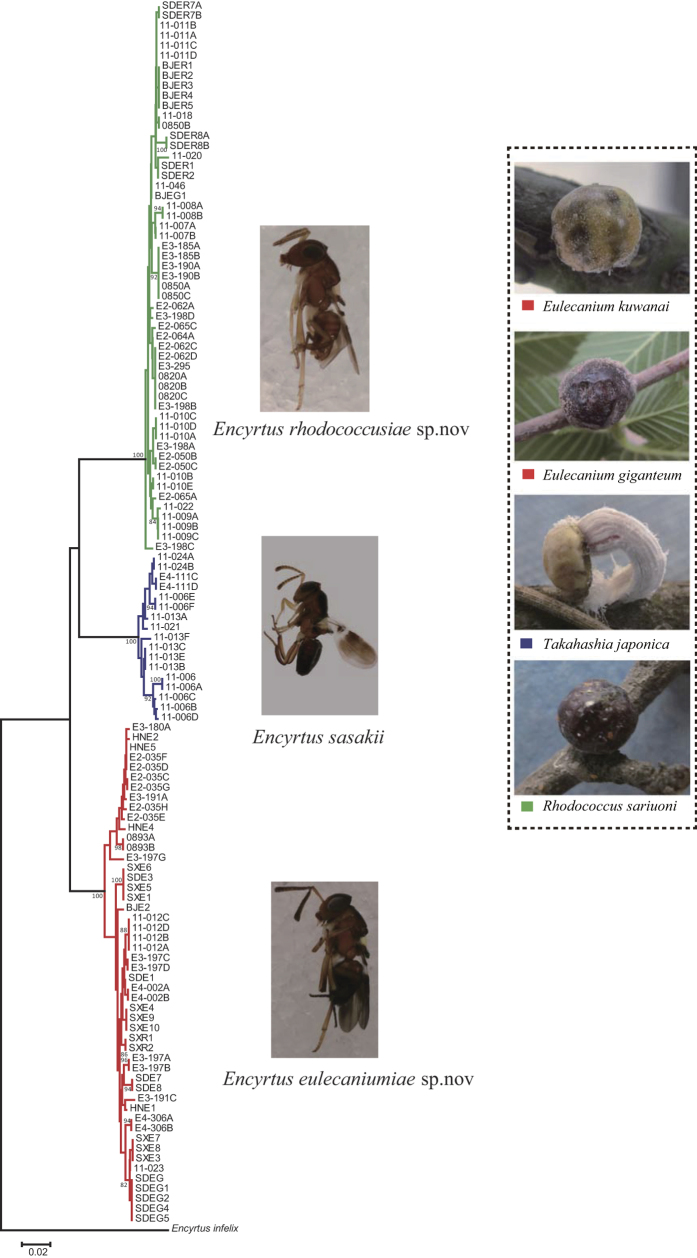
NJ tree based on maximum likelihood analysis of sequences of the COI gene. Bootstrap = 1000, and bootstrap values < 75% are omitted. *Encyrtus infelix* was selected as the outgroup. Colours of branches and letters indicate used hosts (shown on the right site) in China. Photos of *Encyrtus* spp. were taken by author Ying Wang, and photos of soft scale hosts by author Xu-Bo Wang.

An NJ tree was constructed on the basis of COI data in MEGA 6 using the K2P model with 1,000 bootstraps. The NJ analysis revealed three clusters according to the node supports and topologies, and they corresponded to *E. rhodococcusiae*, *E. eulecaniumiae* and *E. sasakii* (Fig. 5). The three species can be easily grouped into three lineages though Bayesian inference analysis, which shows that *E. rhodococcusiae* may be closer to *E. sasakii* than to *E. eulecaniumiae* (Supplementary Figure S2).

### Taxonomy of the Encyrtus species

#### *Encyrtus eulecaniumiae* sp. nov. Wang & Zhang

##### Diagnosis

**Figure 6:**
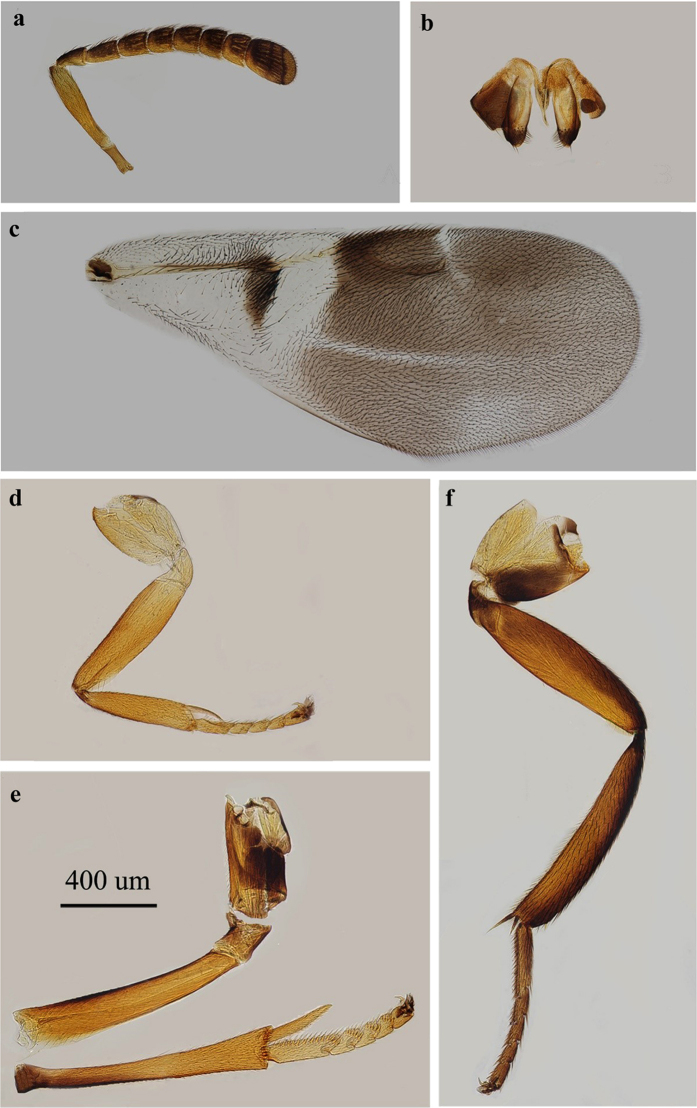
*Encyrtus eulecaniumiae*. (**a**) antenna; (**b**) ovipositor; (**c**) fore wing; (**d**) fore leg; (**e**) mid leg; (**f**) hind leg. Photos were taken by author Ying Wang.

Females of *E. eulecaniumiae* are similar in appearance to those of *E. sasakii* and *E. rhodococcusiae*. Figure 6.

Molecular diagnostic characteristics of *E. eulecaniumiae*. The divergence of mtDNA COI sequences between populations of *E. eulecaniumiae* and *E. sasakii* is approximately 10.04% (K2P model), and that between populations of *E. eulecaniumiae* and *E. rhodococcusiae* is approximately 10.60% (K2P model). The diagnostic characteristics of the COI sequences (GenBank accession numbers KX242564–KX242611) are as follows: 14, A; 47, G; 53, G; 68, G; 134, A; 203, G; 236, G; 266, G; 271, G; 281, A; 341, A; 461, A; 470, A; and 533, A. The diagnostic characteristics of the 28S sequences (GenBank accession numbers KX242678–KX242711) are 217, T and 451, A.

Biological diagnostic characteristics: *E. eulecaniumiae* uses *Eulecanium kuwanai* (Kuwana) and *Eulecanium giganteum* (Shinji) as hosts.

##### Description

(Supplementary Text file S1).

##### Host

Eulecanium kuwanai, E. giganteum (Hemiptera: Coccidae).

##### Distribution

China (Beijing, Heilongjiang, Henan, Inner Mongolia, Shandong, Shanxi) (Supplementary Figure S3).

##### Etymology

The specific epithet is derived from the host of the new species.

##### Holotype

♀: China, Beijing, Shijingshan (Badachu Park), 15. V. 2014, Col. Ying Wang, ex. *Eulecanium kuwanai* on *Ulmus pumila* (on slide of CODE E4-306A, DNA material CODE E4-306A. IZCAS).

##### Paratypes

2♀, Beijing, Shijingshan (Badachu Park), 2014-V-15, Col. Ying Wang, ex. *Eulecanium kuwanai* on *Ulmus pumila*; 4♀♀, 2♂♂, Heilongjiang, Harbin, 2011-VI-9, Col. Ying Wang, ex. *Eulecanium kuwanai* on *Ulmus pumila*; 16♀♀, 5♂♂, Henan, Zhengzhou, 2007-V-4, Col. Xiong Wang, ex. *Eulecanium kuwanai* on *Sophora japonica*; 221♀♀, 72♂♂ Inner Mongolia, Hohhot, 2012-V-26, Col. Haibin Li, ex. Encyrtus giganteum on *Sophora japonica*; 62♀♀, 40♂♂, Shanxi, Taiyuan, 2007-V-1, Col. Jie Li, ex. *Eulecanium kuwanai* on *Sophora japonica*; 85♀♀, 33♂♂, Shandong, Taian, 2008-V-11, Col. Yanzhou Zhang, ex. *Eulecanium kuwanai* on *Sophora japonica*.

Non type material. 3♀♀, Beijing, Haidian, 2008-VI-1, Col. Yanzhou Zhang, ex. *Eulecanium kuwanai* on *Ulmus pumila*; 5♀♀, 2♂♂, Beijing, Haidian (Xiangshan), 2010-VI-1, Col. Yanzhou Zhang, ex. *Eulecanium kuwanai* on *Sophora japonica*; 62♀♀, 14♂♂, Beijing, Haidian, 2012-V-9, Col. Ying Wang, ex. *Eulecanium kuwanai* on *Ulmus pumila*; 4♀♀, Beijing, Haidian (Xiangshan), 2013-V-11, Col. Ying Wang, Linlin Zheng, Xubo Wang, ex. *Eulecanium kuwanai* on *Juglans* sp.; 1♂, Beijing, Haidian, 2014-IV-16, Col. Xubo Wang, Xu Zhang, Yaoguang Qin, ex. *Eulecanium kuwanai* on *Sophora japonica*; 3♀♀, 2♂♂, Beijing, Haidian, 2014-IV-16, Col. Xubo Wang, Xu Zhang, Yaoguang Qin, ex. *Eulecanium kuwanai* on *Koelreuteria paniculata*; 77♀♀, 22♂♂, Heilongjiang, Harbin, 2011-VI-28, Col. Ying Wang, ex. *Eulecanium kuwanai* on *Ulmus pumila*; 43♀♀, 34♂♂, Heilongjiang, Harbin, 2012-VI-1, Col. Xiuwei Liu, ex. *Eulecanium kuwanai* on *Rosa davurica*; 4♀♀, 2♂♂, Heilongjiang, Harbin, 2012-VI-1, Col. Xiuwei Liu, ex. *Eulecanium kuwanai* on *Ulmus pumila*; 80♀♀, 50♂♂, Inner Mongolia, Baotou, 2013-V-22, Col. Xu Zhang, Haibin Li, Xubo Wang, ex. *Eulecanium kuwanai* on *Sophora japonica*; 52♀♀, 41♂♂, Inner Mongolia, Hohhot, 2012-V-26, Col. Haibin Li, ex. *Eulecanium kuwanai* on *Sophora japonica*; 11♀♀, 4♂♂ Inner Mongolia, Hohhot, 2012-V-26, Col. Haibin Li, ex. *Eulecanium* giganteum on *Ulmus pumila*; 2♀♀, 1♂, Shanxi, Linfen, 2007-V, Col. Jie Li, ex. *Eulecanium kuwanai* on *Sophora japonica*; 24♀♀, 10♂♂, Shandong, Taian, Taishan, 2008-V-10, Col. Yanzhou Zhang, ex. *Eulecanium kuwanai* on *Albizzia julibrissn*.

#### *Encyrtus rhodococcusiae* sp. nov. Wang & Zhang

##### Diagnosis

**Figure 7:**
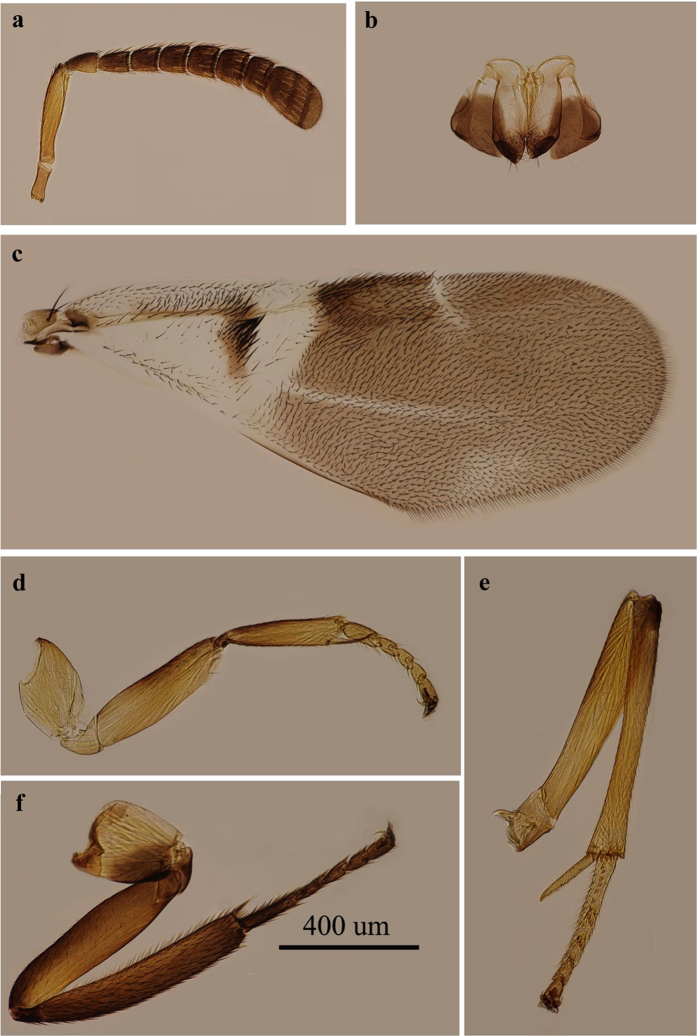
*Encyrtus rhodococcusiae*. (**a**) antenna; (**b**) ovipositor; (**c**) fore wing; (**d**) fore leg; (**e**) mid leg; (**f**) hind leg. Photos were taken by author Ying Wang.

*E. rhodococcusiae* females are similar in appearance to those of *E. sasakii* and *E. eulecaniumiae*. Figure 7.

Molecular diagnostic characters of *Encyrtus rhodococcusiae*. The divergence between the mtDNA COI sequences of populations of *E. rhodococcusiae* and *E. sasakii* is approximately 10.60% (K2P model), and that between populations of *E. rhodococcusiae* and *E. eulecaniumiae* is approximately 10.13% (K2P model). The diagnostic characteristics of the COI sequences (GenBank accession numbers KX242612–KX242666) are as follows: 14, T; 26, A; 102, A; 149, A; 161, C; 176, G; 215, G; 266, T; 269, A; 281, T; 389, A; 446, A; 468, C; 470, T; 521, T; and 530, G. The diagnostic characteristics of the 28S sequences (GenBank accession numbers KX242712–KX242742) are those of 217, C; and 451, G.

Biological diagnostic characteristics: *Encyrtus rhodococcusiae* uses *Rhodococcus sariuoni* as a host.

##### Description

(Supplementary Text file S1).

##### Host

*Rhodococcus sariuoni* (Hemiptera: Coccidae).

##### Distribution

In China, it is recorded as being from Beijing, Heilongjiang, Jilin, Qinghai, Shaanxi, and Shandong provinces (Supplementary Figure S3).

##### Etymology

The specific epithet is derived from the host of the new species.

##### Holotype

♀, Shandong, Linyi, 2011-V-9, Col. Xuemei Yang, ex. *Rhodococcus sariuoni* on *Crataegus pinnatifida* (on slide of CODE 11-009A, DNA material CODE 11-009A. IZCAS).

##### Paratypes

2♀♀, Beijing, Haidian, 2006-V-15, Col. Yanzhou Zhang, ex. *Rhodococcus sariuoni* on *Malus spectabilis*; 3♀♀, Heilongjiang, Harbin, 2007-VI-15, Col. Yanzhou Zhang, ex. *Rhodococcus sariuoni* on *Prunus persica*; 6♀♀, 1♂, Jilin, Changchun, 2011-VI-9, Col. Ying Wang, ex. *Rhodococcus sariuoni* on *Prunus persica*; 2♀♀, 2♂♂, Qinghai, Xining, 2013-VI-28, Col. Haibin Li, Xubo Wang, Xu Zhang, ex. *Rhodococcus sariuoni* on *Prunus cerasifera*; 7♀♀, 2♂♂, Shandong, Taian, 2008-V-9, Col. Yanzhou Zhang, ex. *Rhodococcus sariuoni* on *Prunus cerasifera*; 2♀♀, Shaanxi, Xianyang, 2011-V-9~15, Col. Feng Yuan, ex. *Rhodococcus sariuoni* on *Malus sieversii*.

Non type material. 1♀, Beijing, Haidian, 2008-V-15, Col. Yanzhou Zhang, ex. *Rhodococcus sariuoni* on *Malus spectabilis*; 25♀♀, 3♂♂, Beijing, Haidian, 2011-V-15, Col. Yanzhou Zhang, ex. *Rhodococcus sariuoni* on *Malus spectabilis*; 1♀, Beijing, Mentougou, 2011-VI-4, Col. Yanzhou Zhang, ex. *Rhodococcus sariuoni* on *Malus pumila*; 3♀♀, 3♂♂, Beijing, Mentougou, 2012-V-15, Col. Feng Yuan, Xu Zhang, ex. *Rhodococcus sariuoni* on *Malus spectabilis*; 165♀♀, 57♂♂, Heilongjiang, Harbin, 2012-VI-6, Col. Xiuwei Liu, ex. *Rhodococcus sariuoni* on *Cerasus tomentosa*; 1714♀♀, 620♂♂, Heilongjiang, Harbin, 2012-VI-6, Col. Xiuwei Liu, ex. *Rhodococcus sariuoni* on *Amygdalus triloba*; 3♀♀, Heilongjiang, Harbin, 2012-VI-6, Col. Xiuwei Liu, ex. *Rhodococcus sariuoni* on *Prunus cerasifera*; 2♀♀, 1♂, Heilongjiang, Harbin, 2012-VI-6, Col. Xiuwei Liu, ex. *Rhodococcus sariuoni* on *Ulmus pumila*; 1♀, 1♂, Heilongjiang, Harbin, 2014-V-27, Col. Ying Wang, ex. *Rhodococcus sariuoni* on *Padus racemosa*; 9♀♀, 9♂♂, Heilongjiang, Suihua, 2014-V-28, Col. Ying Wang, ex. *Rhodococcus sariuoni* on *Padus racemosa*; 1♀, Qinghai, Xining, 2007-VI-22, Col. Yanzhou Zhang, ex. *Rhodococcus sariuoni* on *Prunus persica*; 6♀♀, 1♂, Qinghai, Xining, 2013-VI-26, Col. Haibin Li, Xubo Wang, Xu Zhang, ex. *Rhodococcus sariuoni* on *Armeniaca vulgaris*; 2♂♂, Shandong, Taian, 2008-V-16, Col. Yanzhou Zhang, ex. *Rhodococcus sariuoni* on *Prunus cerasifera*; 39♀♀, 8♂♂, Shandong, Taian, 2010-V-16, Col. Jun Deng, Yanzhou Zhang, ex. *Rhodococcus sariuoni* on *Prunus cerasifera*; 14♀♀, 2♂♂, Shandong, Linyi, 2011-V-9, Col. Xuemei Yang, ex. *Rhodococcus sariuoni* on *Crataegus pinnatifida*; 3♀♀, Shandong, Linyi, 2011-IV-30, Col. Xuemei Yang, ex. *Rhodococcus sariuoni* on *Crataegus pinnatifida*; 16♀♀, 1♂, Shandong, Weifang, 2011-V-9, Col. Jun Deng, Yanzhou Zhang, ex. *Rhodococcus sariuoni* on *Prunus persica*; 1♀, Shandong, Weifang, 2011-V-9, Col. Jun Deng, Yanzhou Zhang, ex. *Rhodococcus sariuoni* on *Malus sieversii*; 4♀♀, 1♂, Shandong, Weifang, 2011-V-9, Col. Yanzhou Zhang, ex. *Rhodococcus sariuoni* on *Prunus cerasifera*.

#### *Encyrtus sasakii* Ishii

**Figure 8:**
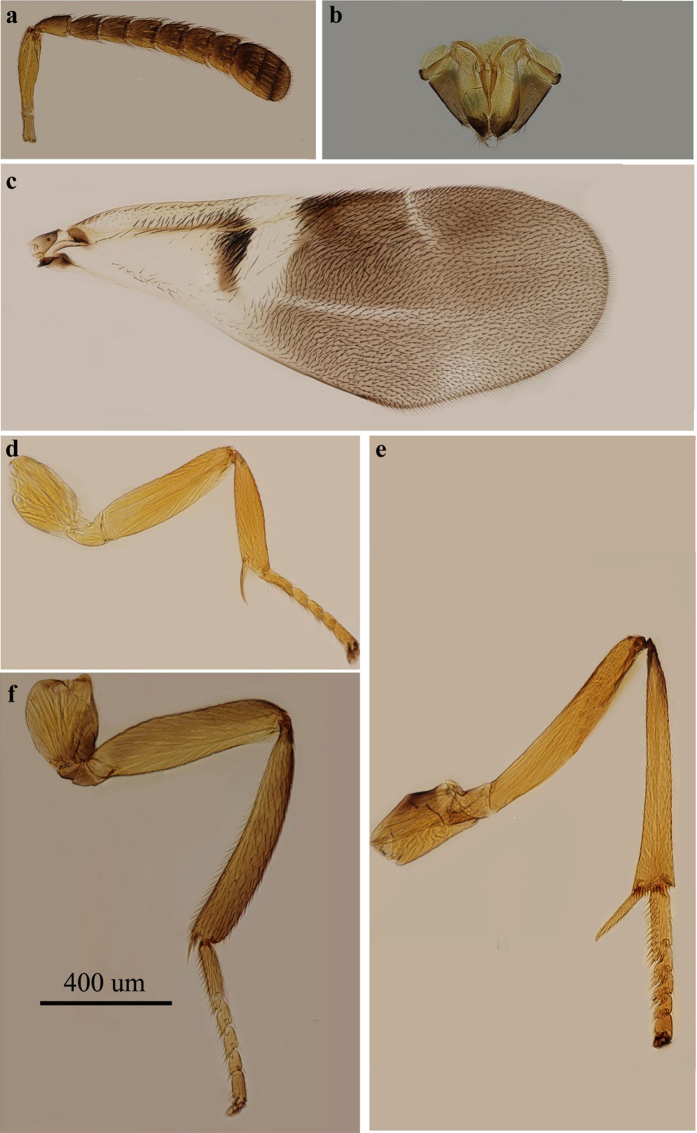
*Encyrtus sasakii*. (**a**) antenna; (**b**) ovipositor; (**c**) fore wing; (**d**) fore leg; (**e**) mid leg; (**f**) hind leg. Photos were taken by author Ying Wang.

*Encyrtus sasakii* Ishii^37^. Holotype ♀, Japan. Figure 8.

##### Diagnosis

*Encyrtus sasakii* females are similar in appearance to those of *Encyrtus eulecaniumiae* and *E. rhodococcusiae*.

Molecular diagnostic characteristics of *Encyrtus sasakii*. The divergence of the mtDNA COI sequences between populations of *E. sasakii* and *E. rhodococcusiae* is approximately 10.60% (K2P model), and that between populations of *E. sasakii* and *E. eulecaniumiae* is approximately 10.04% (K2P model). The diagnostic characteristics of the mitochondrial COI sequences (GenBank accession numbers KX242547–KX242563) are as follows: 14, G; 32, G; 122, A; 224, G; 227, G; 233, A; 245, G; 254, A; 266, A; 271, A; 281, G; 284, T; 290, G; 320, G; 335, A; 431, G; 443, A; 467, A; 470, G; and 494, G; and for nuclear 28S sequences (GenBank accession numbers KX242667–KX242677), they were 217, T; and 451, G.

Biological diagnostic characteristics: *Encyrtus sasakii* is hosted by *Takahashia japonica*.

##### Description

(Supplementary Text file S1).

##### Host

*Takahashia japonica* (Hemiptera). The recorded host *Kermes* sp. needs confirmation.

##### Distribution

It is recorded as being from Anhui, Jiangsu, Jiangxi, and Zhejiang provinces in China (Supplementary Figure S3) and seems to be restricted to subtropical areas. It is also distributed in Japan.

##### Material examined

12♀♀, 1♂, Jiangsu, Nanjing, 2011-IV-16, Col. Liang Ding, ex. *Takahashia japonica* on *Albizzia julibrissn*; 1♀, Jiangsu, Nanjing, 2011- IV-26, Col. Liang Ding, ex. *Takahashia japonica* on *Albizzia julibrissn*; 5♀♀, 3♂♂, Jiangsu, Nanjing, 2011- IV-28, Col. Liang Ding, ex. *Takahashia japonica* on *Albizzia julibrissn*; 1♀, 1♂, Jiangsu, Nanjing, 2011-V-7, Col. Liang Ding, ex. *Takahashia japonica* on *Albizzia julibrissn*; 4♀♀, Anhui, Wuhu, 2011-IV-27, Col. Huizi Ye, ex. *Takahashia japonica* on *Robinia pseudoacacia*; 14♀♀, Zhejiang, Ningbo, 2011-IV-29~2011-V-9, Col. Tongxin Zhang, ex. *Takahashia japonica* on *Lorpetalum chinense*; 4♀♀, Jiangxi, Nanchang, 2014-IV-21, Col, Jun Deng & Yanzhou Zhang, *Takahashia japonica* on *Acer buergerianum*.

## Discussion

### Traditional taxonomy and cryptic species

Morphological data have traditionally been used to delimit species, and the morphology-based taxonomy is still the most important discipline for assigning taxonomically valid names on the basis of typing specimen(s)^12^,^38^. Thus, a thorough morphological study of cryptic species is necessary before nomenclature and description. This process will not only provide us with a better estimate for species delimitation by comparing with other sources of data but will also undoubtedly help us to understand the morphological processes of speciation and to discover the characteristics upon which natural selection acts^16^. The quantitative and statistical analyses of morphological variations in cryptic species should reduce the subjectivity of traditional taxonomy^39^,^40^,^41^. In the current study, though nearly all the morphological characteristics of cryptic species are continuous and/or overlapping (a morphological character distribution trait that may be present in all cryptic species), morphometric analysis shows that some characteristics, such as F1W and F2L (Fig. 1), display significant differences between cryptic species and provide a taxonomic background to distinguish among them. It is also worth mentioning that the two characters F3W and F5W are significantly different among the cryptic species, and both antenna components are important organs in the host location and mating processes of parasitoids^42^. The significant difference between F3W and F5W is probably related to the diversification of biological elements (such as host use) and genetic markers. Such significant differences in different morphological traits were also found for cryptic species in other animal groups^43^,^44^,^45^.

Although the scatterplot of the shape PCA through MRA was barely satisfactory in this study (Fig. 2a), it did indicate that divergence of the morphological characters among the cryptic species has occurred, particularly when analysing the most discriminating ratios between the cryptic species (Fig. 2a). The LDA ratio extractor may help in the search for good characteristics for the diagnosis of other species^46^,^47^,^48^. However, in the present study, this extractor did not generated any characteristic that can be reliably to separate these three cryptic species (Table 2), and there is still a possibility that distinguishing morphological characters are not addressed in these cryptic species.

GMA have been used to study various insect taxa, ranging from the species level to superfamily analysis^49^,^50^, and it was proven to be useful for the delimitation of cryptic taxa in various arthropod groups^51^,^52^,^53^. Similar to MRA, GMA helped us to understand the variations of the forewing in cryptic species that may be newly speciated, but it did not reveal significantly different characteristics for species diagnosis.

### Molecular analyses

Molecular analyses revealed three distinct clusters that corresponded to species delineation based on biological characteristics (i.e., host range and mating tests). These results are consistent with the study by Chesters *et al*.^38^ that was based on a smaller sample size. Although many cryptic species have been found by using mitochondrial DNA barcodes (COI), we found that 28S-D2 nuclear rDNA sequences are useful for identifying these three cryptic species (with only one bp of change, but stable), and the diagnosis characteristics are also used in their description. Nuclear genes (such as 28S) are thought to be less sensitive for detecting recent speciation events when compared with mtDNA genes, although they have been used for distinguishing species^54^,^55^,^56^,^57^. The present study shows that 28S-D2 could be an important marker for the identification of cryptic species, although more groups of cryptic species should be tested. Once the researcher reaches a conclusion concerning species differences, DNA barcoding data can be used afterwards to more easily indicate the differences either using tree-, distance- or character-based methods as performed in the present study. The frequency with which cryptic species are uncovered with DNA sequence data suggests that molecular data should be incorporated into the routine taxonomy procedure, particularly when the research indicates that morphological analysis can take us no further^24^,^25^. Moreover, descriptions of each species should include the molecular features that are actually diagnostic of the species^58^,^59^. Genetic material (including that of the Holotype if possible) should be preserved as a reference material so that subsequent molecular analysis is possible for future studies.

### Biological elements

In our study, the host species was directly related to the three species of the *E. sasakii* complex. The individuals of *E. eulecaniumiae* were all reared from *Eulecanium kuwanai* and *Eulecanium giganteum*. *E. rhodococcusiae* were all reared from *Rhodococcus sariuoni*, and *E. sasakii* was reared from *Takahashia japonica*. Given the high host specificity revealed by these three species, each was collected within the distribution of their hosts. *E. eulecaniumiae* is usually distributed over northern China, and it closely overlaps the *E*. *rhodococcusiae* distribution. In contrast, *E. eulecaniumiae* is parapatric with *E. sasakii*. In many cases, additional data, such as the host range, geographic locations or mating tests, might also help to distinguish among cryptic species^9^,^10^,^36^,^60^,^61^,^62^, and such information should also be incorporated into the description.

## Conclusions

The present study demonstrated that integrating multiple data resources in taxonomy, particularly when associated with formal species description, is a practical way to improve the quality of species hypotheses and their associated descriptions. The study also highlights the difficulty in drawing objective morphological limits between cryptic species. Although the morphological characteristics are highly similar and continuous and/or overlapping for cryptic species, such as *Encyrtus sasakii*, *E. eulecaniumiae* and *E. rhodococcusiae*, statistical analyses show that some morphological characteristics have significantly differentiated, which is congruent with the results generated from molecular and biological data. Molecular character tools (DNA barcoding) are powerful for the identification and delimitation of cryptic species, and thus the molecular characteristics should be provided in their description and diagnosis. It is also worth emphasizing that multiple sources of diagnosis, including biological and possibly reproductive information, are important during the process of identifying cryptic species.

## Materials and Methods

### Host population collection and data acquisition

From 2006 to 2014, we surveyed soft scale hosts and their *Encyrtus* parasitoid wasps in mainland China (Supplementary Figure S3). The rearing and preservation of parasitoids was performed in the same way as by Chesters *et al*.^36^. Representative specimens of the *Encyrtus* complex were used in the MRA, geometric morphometric analysis and molecular sequencing. Female specimens were randomly sampled from different populations according to the population size. The head (with antennae), the wings and the gaster were removed from the body, mounted on slides and uniquely coded; the thorax with the same code was used for DNA extraction and then mounted on the same slide for morphological study. Soft scale insects were identified by Prof. San-An Wu (Beijing Forestry University).

### Morphometric analysis (GMA)

We measured the morphometric variables in female specimens using an eyepiece micrometre under a Leitz Dialux 20 (Ernst Leitz, GMBH, Wetzler, Germany) microscope. Measurements were taken in accordance with Noyes^63^ and Wang *et al*.^64^. All the chosen characters (Table 1) are considered diagnostic and have frequently been used in Encyrtidae taxonomy^65^,^66^.

Boxplots were made to show the distribution of measurements for each character. Post-hoc comparisons of each morphological character were conducted using Tukey’s HSD test at a 95% family-wise confidence level. All statistical analyses and plots were obtained with R^67^.

### Multivariate ratio analysis (MRA)

We performed a shape principal component analysis (shape PCA) and plotted its isometric size against the first two shape PCs. We also calculated the PCA ratio spectrum for each shape PC as well as the allometry ratio spectrum. The PCA ratio spectrum is a graphical tool that aims to interpret the principal components of shape in terms of body ratios. In a similar manner, the allometry ratio spectrum reveals the allometric behaviour of the ratios. The linear discriminant analysis (LDA) ratio extractor reveals the best ratios for separating two or more groups with the help of an LDA. The measurements were calculated to determine how much of the total difference was due to the size and how much was due to the shape. These methods allow for the interpretation of the results of the frequently used PCA and LDA by body ratios, which can then be directly incorporated into species identification diagnostics. MRAs were implemented using R^67^ with scripts provided by Baur & Leuenberger^34^ (under ‘File S1’) and by Baur *et al*.^48^ Scatterplots were generated with the package ‘ggplot2’^68^.

### Geometric morphometric analysis (GMA)

**Figure 9:**
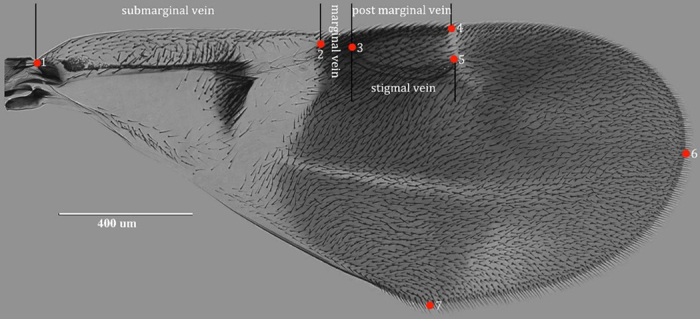
Photograph of forewing (*Encyrtus eulecaniumiae* sp. nov., Sample ID: 11-012A) marked by seven landmarks. Photo taken by author Ying Wang. The number of landmarks are as follows: (1), the beginning of the submarginal vein; (2), the ending of the submarginal vein/ beginning of the marginal vein; (3), the ending of the marginal vein/ beginning of he post marginal vein/ beginning of the stigma vein; (4), the ending of the postmarginal vein; (5), the ending of the stigma vein; (6), the tip of the fore wing; (7), and the tip of the posterior margin of the fore wing.

Photomicrographs were acquired from slide-mounted specimens using an EVOS f1 inverted microscope (AMG, U.S.A.). Seven landmarks were selected to describe the variations in wing morphology (Fig. 9). The landmarks were as follows: 1, beginning of submarginal vein; 2, end of submarginal vein/beginning of marginal vein; 3, end of marginal vein/beginning of post marginal vein/beginning of stigmal vein; 4, end of postmarginal vein; 5, end of stigma vein; 6, tip of forewing; and 7, tip of posterior margin of forewing. The Cartesian coordinates of the landmarks were digitized with tps-DIG 2.05^69^.

We first performed a generalized least squares Procrustes analysis on data acquired from tps-DIG 2.05, and we quantified the similarity in covariance structures at different levels. Next, we computed the matrix correlations between the corresponding covariance matrices. The matrix correlations were tested with a matrix permutation test that was adapted for geometric morphometrics by using PCA with 10,000 permutations. All analyses were performed in MorphoJ^70^.

### Genetic analyses

For DNA extraction, amplification and sequencing, we followed the protocols described by Chesters *et al*.^36^. Forward and reverse sequences were assembled and reciprocally edited with BioEdit v7.1.3.0^71^. Neighbour-joining (NJ) tree reconstruction was performed by MEGA6^72^ using the Kimura 2-parameter (K2P) model^73^. Genetic distances were calculated using both K2P distances and the uncorrected *p*-distance. Bayesian analyses using COI sequences were conducted with MrBayes v3.2^74^ consisting of two Markov chain Monte Carlo (MCMC) analyses run for 4 000 000 generations, sampling trees every 100 generations and using four chains and default priors. The convergence between the two runs was assessed using the average standard deviation of the split frequencies (below 0.01). The two runs were combined after the removal of the first 1 000 000 generations from each run as a burn-in. The HKY + I + G model of evolution for the COI gene was estimated using jModelTest v2.1.3^75^, and it was selected on the basis of Akaike information criterion (AIC), as suggested by Posada and Buckley^76^. The identification of diagnostic characters within COI sequences was performed in the Characteristic Attribute Organization System (CAOS)^77^,^78^. When all members of a species share these characters, they are termed ‘simple pure characters’^77^.

## Additional Information

**How to cite this article**: Wang, Y. *et al*. Formal nomenclature and description of cryptic species of the *Encyrtus sasakii* complex (Hymenoptera: Encyrtidae). *Sci. Rep*. **6**, 34372; doi: 10.1038/srep34372 (2016).

## Supplementary Material

Supplementary Information

## Figures and Tables

**Table 1:** The morphological characters and their abbreviations as measured for the morphometric study.

Abbreviations	Characters
CL	The length of the clava
CW	The maximum width of the clava
F1L	The length of the funicle, segment one
F1W	The maximum width of the funicle, segment one
F2L	The length of the funicle, segment two
F2W	The maximum width of the funicle, segment two
F3L	The length of the funicle, segment three
F3W	The maximum width of the funicle, segment three
F4L	The length of the funicle, segment four
F4W	The maximum width of the funicle, segment four
F5L	The length of the funicle, segment five
F5W	The maximum width of the funicle, segment five
F6L	The length of the funicle, segment six
F6W	The maximum width of the funicle, segment six
FT	The length of the fore tibia
HT	The length of the hind tibia
MT	The length of the mid tibia
MTS	The length of the mid tibial spur
OV	The length of the ovipositor.
PL	The length of the pedicel
PVL	The length of the postmarginal vein.
PW	The maximum width of the pedicel
SL	The length of the scape
SVL	The length of the stigma vein.
SW	The maximum width of the scape

**Table 2:** First- and second-best ratios found by the LDA ratio extractor for separating the females of three *Encyrtus* species.

Group comparison	Best ratios	Range group 1	Range group 2	Standard distance	δ
*E. sasakii vs. E. eulecaniumiae*	*F2L/PVL	0.28–0.40	0.23–0.34	4.26	0.03
	F5W/OV	0.25–0.35	0.29–0.36	3.87	0.03
*E. sasakii vs. E. rhodococcusiae*	*F5W/OV	0.25–0.35	0.27–0.40	4.92	0.04
	SL/HT	0.36–0.44	0.33–0.44	4.36	0.04
*E. eulecaniae vs. E. rhodococcusiae*	SL/F1W	3.43–5.64	3.21–5.18	3.13	0.02
	F1L/F3L	1.00–1.47	0.93–1.38	2.55	0.03
*E. sasakii vs*.(*E. eulecaniumiae + E. rhodococcusiae*)	F5W/OV	0.25–0.35	0.27–0.40	4.35	0.04
	F2L/SVL	0.21–0.31	0.21–0.35	3.87	0.05
*E. eulecaniumiae vs*.(*E. sasakii + E. rhodococcusiae*)	F6L/PVL	0.19–0.29	0.20–0.34	2.41	0.01
	F1W/CW	0.39–0.64	0.43–0.61	2.18	0.01
*E. rhodococcusiae vs*.(*E. sasakii + E. eulecaniumiae*)	F1L/F1W	1.00–1.47	1.20–1.85	2.89	0.04
	F5W/OV	0.27–0.40	0.25–0.36	2.28	0.05

^*^Ratios with less overlapping.

**Table 3:** Genetic distance of three cryptic species of *Encyrtus* using COI haplotype data (Mean ± SE).

	*E. sasakii*	*E. eulecaniumiae*	*E. rhodococcusiae*
*E. sasakii*	1.24% ± 0.30%/1.23% ± 0.26%	9.31% ± 1.11%	9.84% ± 1.03%
	0.00~2.38%/0.00~2.33%	8.26%~10.41%	8.98%~10.77%
*E. eulecaniumiae*	10.04% ± 1.28%	1.74% ± 0.32%/1.70% ± 0.31%	9.38% ± 1.11%
	8.81%~11.36%	0.00~3.33%/0.00~3.23%	8.62%~10.23%
*E. rhodococcusiae*	10.60% ± 1.30%	10.13% ± 1.30%	0.96% ± 0.21%/0.95% ± 0.21%
	9.59%~11.72%	9.22%~11.16%	0.00~2.38%/0.00~2.33%

(K2P distances are shown under the diagonal, and *p* distances are above the diagonal; grey shading indicates intra-specific divergences, the values on the left are K2P distances, and the values on the right are *p* distances).

**Table 4:** Character-based DNA barcodes (COI) for three cryptic species of *Encyrtus*.

Position	14	26	32	47	53	68	102	122	134	149	
*E. sasakii*	**G**	G	**G**	T	A	G	G	**A**	G	T	
*E. eulecaniumiae*	**A**	G	A	**G**	**G**	**A**	G	T	**A**	T	
*E. rhodococcusiae*	**T**	**A**	A	T	A	G	**A**	T	G	**A**	
Position	161	176	203	215	224	227	233	236	245	254	
*E. sasakii*	T	A	A	A	**G**	**G**	**A**	A	**G**	**A**	
*E. eulecaniumiae*	T	A	**G**	A	A	A	T	**G**	A	T	
*E. rhodococcusiae*	**C**	**G**	A	**G**	A	A	T	A	A	T	
Position	266	269	271	281	284	290	320	335	341	389	
*E. sasakii*	**A**	T	**A**	**G**	**T**	**G**	**G**	**A**	T	G	
*E. eulecaniumiae*	**G**	T	T/**G**	**A**	A	A	A	T	**A**	G	
*E. rhodococcusiae*	**T**	**A**	T	**T**	A	A	A	T	T	**A**	
Position	431	443	446	461	467	468	470	494	521	530	533
*E. sasakii*	**G**	**A**	G	G	**A**	T	**G**	**G**	A	A	G
*E. eulecaniumiae*	A	G	G	**A**	G	T	**A**	A	A	A	**A**
*E. rhodococcusiae*	A	G	**A**	G	G	**C**	**T**	A	**T**	**G**	G

Pure diagnostic characters for discriminating all the individuals at 41 selected nucleotide positions for COI except at position 271 for *E. eulecaniumiae* are shown in bold.
