# Supplementary material for: Formal nomenclature and description of cryptic species of the Encyrtus sasakii complex (Hymenoptera: Encyrtidae)
Source: Sci Rep. 2016 Oct 4;6:34372. doi: 10.1038/srep34372 (PMC5048151; doi:10.1038/srep34372)
Supplement: Supplementary Information [file srep34372-s1.pdf]

Formal nomenclature and description of cryptic species of the *Encyrtus sasakii* complex (Hymenoptera: Encyrtidae)

Ying Wang[1], Qing-Song Zhou[1,2], Hui-Jie Qiao[1], Ai-Bing Zhang[3], Fang Yu[1,2], Xu-Bo Wang[4], Chao-Dong Zhu[1,2], Yan-Zhou Zhang[1,2]*

1 Key Laboratory of Zoological Systematics and Evolution, Institute of Zoology, Chinese Academy of Sciences, Beijing 100101, China

2 University of Chinese Academy of Sciences (UCAS), No. 19A Yuquan Road, Beijing 100049, China

3 College of Life Sciences, Capital Normal University, Beijing 100048, China

4 Key Laboratory for Silviculture and Conservation of Ministry of Education, Beijing Forestry University, Beijing 100083, China

* *Corresponding author E-mail: zhangyz@ioz.ac.cn*

Authors Ying Wang and Qing-Song Zhou contributed equally to this study.

Text File S1

Description of *Encyrtus* species

***Encyrtus eulecaniumiae* sp. nov. Wang & Zhang, Figure 6**

Description.

Female (Holotype): Length about 3 mm including ovipositor sheaths. Head black in ocellar area, from anterior ocellus to top of scrobes black to brown yellow, and malar space area brown; face largely brown yellow; radicle brown mixed pale orange; scape (Figure 6a) mostly pale brown; pedicel dark brown or black; funicle with segments black; clava black; pronotum dark brown, laterally brown; thorax clothed in setae which dark brown, mesoscutum mostly black, laterally brown, sctuellum black but a broad transverse yellow band on the scutellum, with a tuft of black bristles on the tip; metanotum black; tegula brown; mesopleuron pale brown; fore and hind coxae brown yellow; mid coxa mostly brown;legs otherwise brown; basal one third of fore wing hyaline, and the others infuscate, fore wing with a group of long bristles just below the apical third of the submarginal; propodeum brown dorsally, yellowish brown laterally; gaster black.

Head. In dorsal view, frontovertex about half head width; ocelli forming an obtuse angle about 120°; in front view, scrobal depression ∩-shaped; eye at least superficially bare; torulus separated from mouth margin by about one its own length; torulus separated from each other by about 4× own diameter. Antenna (Figure 6a) 13-segmented, scape cylindrical and 4.5× as long as broad; but approximate ratio of

segments from pedicel = 101: 98: 85: 81: 82: 78: 76: 87: 56: 47; F1 about 1.4× longer than wide, F2 a little longer than wide, F3 and F4 as long as wide, the last two a little wider than long; clava compact 3-segmented with transverse sutures; clypeus with three to six of conspicuous, long, suberect setae; maxillary and labial palpi 4 and 3 segmented respectively.

Thorax. Pronotum in dorsal view about one-sixth length of mesoscutumwith surface sloping from posterior margin, uniformly setose and fine reticulate sculpture. Mesoscutum about 1.47× wider than long; uniformly convex, setose and fine reticulate sculpture, without notauli. Scutellum with a tuft of black bristles on the tip. Side of propodeum more or less naked below spiracle, but with a few inconspicuous, translucent setae on posterior margin above hind coxa. mesotibia with strong setae apically, but without differentiated rows of spines, and with mesotibial spur only about 1.7× as long as apical width of tibia (Figure 6e); each leg with basitarsus robust and longest, the others equal in size; the basitarsus of mesotarsus about 2× as long as wide and about as long as remaining segments. Fore wing (Figure 6c) hyaline and distinctly infuscate in the apical two-thirds; a fuscous dot below the apical two-third of the submarginal vein and robust bristle on the fuscous area. SM: MV: STV: PMV = 72:5:25:23; stigmal vein apically curved.

Gaster. Gaster very broadly sessile, with seven visible, uniformly gastral tergites; hypopygium very nearly reaching apex of gaster. Ovipositor sheaths yellow, apex one-third dark brown (Figure 6b).

Relative measurements: HW 96, FV 46, POL, 33, AOL 19, OOL 6, OCL 7, POD 5,

AOD 4, EL 58, EW 38, MS 30, SL 32, SW 7, FWL 204, FWW 83; HWL 169, HWW 53, OL 34, [MT 91]

Male: generally similar to female but for antennal structure and genitalia; yellow band on the scutellum obviously smaller than female.

***Encyrtus rhodococcusiae* sp. nov. Wang & Zhang, Figure 7**

Description.

Female (Holotype): Length including ovipositor 1.9 mm. Head black in ocellar area, frontovertex black; malar space area brown; antenna with scape yellow-brown; pedicel and flagellum dark brown; maxillary and labial palpi yellow-brown; pronotum dark brown to black, laterally brown; thorax clothed in setae which dark brown, mesoscutum mostly black, laterally brown, sctuellum black but a broad transverse yellow band on the scutellum, with a tuft of black bristles on the tip; metanotum dark brown; tegula dark brown; mesopleuron pale brown; fore and hind coxae pale brown yellow (Figure 7d, f); mid coxa mostly brown (Figure 7e); legs otherwise brown; basal one third of fore wing hyaline, and the others infuscate, fore wing with a group of long bristles just below the apical third of the submarginal; propodeum brown dorsally, yellowish brown laterally; gaster black.

Head. In dorsal view, frontovertex about half head width; ocelli forming an obtuse angle about 120°; in front view, scrobes quite shallow and ∩-shaped; eye at least superficially bare; torulus separated from mouth margin by about one its own length; torulus separated from each other by about 2.5× own diameter; antenna with scape

subcylindrical, 3.4× as long as broad; pedicel subtriangular, as broad as scape, 1.4× as long as broad. Antenna (Figure 7a) 13-segmented, scape cylindrical but approximate ratio of segments from pedicel = 70: 65: 55: 61:57: 55: 50: 64: 37: 32; ratio of length and width for each funicle segment: 1.3, 1, 1.02, 0.84, 0.75, 0.63; clava 3-segmented similar to *E. eulecaniumiae*, its apex with a distinctly truncation; mandible plow shaped; clypeus also with three to six conspicuous, long, suberect setae; maxillary and labial palpi 4 and 3 segmented respectively.

Thorax. Mesoscutum about 1.44× wider than long; uniformly convex, setose and fine reticulate sculpture, without notauli. Pronotum very short, about one eleventh as long as mesoscutum, with polygonal reticulate sculpture; scutellum about 1.2× as long as broad, sculpture anteriorly similar to that of mesoscutum; fore wing (Figure 7c) about 2.3× as long as broad; costal cell with more than one line of setae dorsally; SM: MV: STV: PMV = 85:5:35:32; stigma vein apically curved.

Gaster. Hypopygium very nearly reaching apex of gaster. Ovipositor sheaths yellow, apex one-third dark brown (Figure 7b).

Relative measurements: HW 93, FV 46, POL, 33, AOL 19, OOL 8, OCL 5, POD 7, AOD 6, EL 50, EW 30, MS 31, SL 31, SW 9, FWL 162, FWW 61; HWL 116, HWW 35,OL28, [MT 69]

Male: generally similar to female but for antennal structure and genitalia; yellow band on the scutellum obviously smaller than female.

Encyrtus sasakii Ishii, Figure 8

Description.

Female: Length including ovipositor 2.5 mm. Ocellar area and frontovertex black; malar space area brown; antenna with scape yellow-brown; pedicel and flagellum in dorsal aspect dark brown; maxillary and labial palpi yellow-brown; pronotum black, only laterally brown; thorax clothed in setae which black, mesoscutum mostly black, laterally brown, sctuellum black but a broad transverse yellow band on the scutellum, with a tuft of black bristles on the apex of scutellum; metanotum dark brown; tegula dark brown; mesopleuron brown; fore coxa pale brown (Figure 8d), mid and hind coxae mostly brown (Figure 8e, f), legs otherwise brown; basal one third of fore wing hyaline, and the others infuscate, fore wing with a group of long bristles just below the apical third of the submarginal; propodeum brown dorsally, yellowish brown laterally; gaster black.

Head. In dorsal view, frontovertex about half head width; ocelli forming an obtuse angle about 120°; in front view, scrobes broadly ∩-shaped; torulus separated from mouth margin very slightly less than its own legth; torulus separated from each other by about 2.0× own diameter; antenna with scape subcylindrical, 4.7× as long as broad; pedicel subtriangular, as broad as scape, 1.7× as long as broad. Antenna (Figure 8a) 13-segmented, scape cylindrical but approximate ratio of segments from pedicel= 85: 90: 81: 75:73: 64: 65: 65: 50: 44; ratio of length and width for each funicle segment: 1.55, 1.25, 1.07, 0.97, 0.77, 0.72; clava 3-segmented, its apex with a distinctly truncation; mandible plow shaped; clypeus also with four conspicuous, long, suberect

setae; maxillary and labial palpi 4 and 3 segmented respectively.

Thorax. Mesoscutum about 1.60× wider than long; uniformly convex, setose and fine reticulate sculpture, without notauli. Pronotum very short, about one fifth as long as mesoscutum, with polygonal reticulate sculpture; scutellum about 1.23× as long as broad, sculpture anteriorly similar to that of mesoscutum; fore wing (Figure 8c) about 2.44× as long as broad; costal cell with more than one line of setae dorsally; SM: MV: STV: PMV = 96:7:37:37; stigmal vein apically curved.

Gaster. Hypopygium nearly reaching apex of gaster. Ovipositor sheaths yellow, apex one-third dark brown (Figure 8b).

Relative measurements: HW 101, FV 48, POL, 40, AOL 24, OOL 7, OCL 8, POD 6, AOD 8, EL 60, EW 41, MS 30, SL 40, SW 8, FWL 230, FWW 94; HWL 170, HWW 69, OL37 [MT 102] .

Male: generally similar to female but for antennal structure and genitalia; yellow band on the scutellum obviously smaller than female.

Table S1. List of collection message including host, host plant of the specimens used in the study

(collectors's names are abbreviated as follow: Liang Ding=LD; Huizi Ye=HZY; Tongxin Zhang=TXZ; Ju Deng=JD; Yanzhou Zhang=YZZ; Ying Wang=YW; Xiuwei Liu=XWL; Haibin Li=HBL; Xubo Wang=XBW; Xu Zhang=XZ; Jie Li=JL; Xiong Wang=XW; Linlin Zheng=LLZ; Xuemei Yang=XMY; Feng Yuan=FY.)

Code	Old Code	Morphology	Geomorphometric	Location	Collection date	Collector	Host	Host plant	COI	28S
11-006		+	+	Jiangsu,Nanjing	2011-v-7	LD	*Takahashia japonica*	*Albizzia julibrissn*	+	
11-006A	JS06A	+	+	Jiangsu,Nanjing	2011-iv-28	LD	*Takahashia japonica*	*Albizzia julibrissn*	+	+
11-006B	JS06B	+	+	Jiangsu,Nanjing	2011-iv-16	LD	*Takahashia japonica*	*Albizzia julibrissn*	+	+
11-006C	JS06C	+	+	Jiangsu,Nanjing	2011-iv-16	LD	*Takahashia japonica*	*Albizzia julibrissn*	+	+
11-006D	JS06D	+	+	Jiangsu,Nanjing	2011-iv-16	LD	*Takahashia japonica*	*Albizzia julibrissn*	+	+
11-006E		+	+	Jiangsu,Nanjing	2011-iv-16	LD	*Takahashia japonica*	*Albizzia julibrissn*	+	
11-006F		+	+	Jiangsu,Nanjing	2011-iv-16	LD	*Takahashia japonica*	*Albizzia julibrissn*	+	
11-006H		+		Jiangsu,Nanjing	2011-iv-16	LD	*Takahashia japonica*	*Albizzia julibrissn*		
11-006I		+		Jiangsu,Nanjing	2011-iv-16	LD	*Takahashia japonica*	*Albizzia julibrissn*		
11-006J		+	+	Jiangsu,Nanjing	2011-iv-16	LD	*Takahashia japonica*	*Albizzia julibrissn*		
11-006L		+		Jiangsu,Nanjing	2011-iv-16	LD	*Takahashia japonica*	*Albizzia julibrissn*		
11-006M		+		Jiangsu,Nanjing	2011-iv-16	LD	*Takahashia japonica*	*Albizzia julibrissn*		
NJ-TA-001		+	+	Jiangsu,Nanjing	2011-iv-28	LD	*Takahashia japonica*	*Albizzia julibrissn*		
NJ-TA-002		+	+	Jiangsu,Nanjing	2011-iv-16	LD	*Takahashia japonica*	*Albizzia julibrissn*		
11-021		+	+	Jiangsu,Nanjing	2011-iv-26	LD	*Takahashia japonica*	*Albizzia julibrissn*	+	+
11-021C		+	+	Jiangsu,Nanjing	2011-iv-16	LD	*Takahashia japonica*	*Albizzia julibrissn*		
11-024A		+	+	Anhui, Wuhu	2011-iv-27	HZY	*Takahashia japonica*	*Robinia pseudoacacia*	+	+
11-024B		+	+	Anhui, Wuhu	2011-iv-27	HZY	*Takahashia japonica*	*Robinia pseudoacacia*	+	+
11-024C		+	+	Anhui, Wuhu	2011-iv-27	HZY	*Takahashia japonica*	*Robinia pseudoacacia*		
11-013A	JS013A	+	+	Zhejiang, Ningbo	2011-iv-29	TXZ	*Takahashia japonica*	*Lorpetalum chinense*	+	+
11-013B	JS013B	+	+	Zhejiang, Ningbo	2011-iv-29	TXZ	*Takahashia japonica*	*Lorpetalum chinense*	+	
11-013C	JS013C	+	+	Zhejiang, Ningbo	2011-iv-29	TXZ	*Takahashia japonica*	*Lorpetalum chinense*	+	+
11-013E	11-013E	+		Zhejiang, Ningbo	2011-iv-29	TXZ	*Takahashia japonica*	*Lorpetalum chinense*	+	+
11-013F	11-013F	+		Zhejiang, Ningbo	2011-iv-29	TXZ	*Takahashia japonica*	*Lorpetalum chinense*	+	+
11-013H		+	+	Zhejiang, Ningbo	2011-v-9	TXZ	*Takahashia japonica*	*Lorpetalum chinense*		
11-013I		+	+	Zhejiang, Ningbo	2011-v-9	TXZ	*Takahashia japonica*	*Lorpetalum chinense*		
11-013J		+		Zhejiang, Ningbo	2011-v-9	TXZ	*Takahashia japonica*	*Lorpetalum chinense*		
11-013K		+		Zhejiang, Ningbo	2011-v-9	TXZ	*Takahashia japonica*	*Lorpetalum chinense*		
11-013L		+	+	Zhejiang, Ningbo	2011-v-9	TXZ	*Takahashia japonica*	*Lorpetalum chinense*		
11-013M		+	+	Zhejiang, Ningbo	2011-v-9	TXZ	*Takahashia japonica*	*Lorpetalum chinense*		
ZJ-T		+		Zhejiang, Ningbo	2011-iv-29	TXZ	*Takahashia japonica*	*Lorpetalum chinense*		
E4-111C		+		Jiangxin, Nanchang	2014-iv-21	DJ, ZYZ	*Takahashia japonica*	*Acer buergerianum*	+	
E4-111D		+		Jiangxin, Nanchang	2014-iv-22	DJ, ZYZ	*Takahashia japonica*	*Acer buergerianum*	+	
11-012A	HJ012A	+	+	Heilongjiang, Harbin	2011-vi-9	YW	*Eulecanium kuwanai*	*Ulmus pumila*	+	+
11-012B	HJ012B	+	+	Heilongjiang, Harbin	2011-vi-9	YW	*Eulecanium kuwanai*	*Ulmus pumila*	+	+
11-012C	HJ012C			Heilongjiang, Harbin	2011-vi-9	YW	*Eulecanium kuwanai*	*Ulmus pumila*	+	
11-012D	HJ012D			Heilongjiang, Harbin	2011-vi-9	YW	*Eulecanium kuwanai*	*Ulmus pumila*	+	
E3-191A		+	+	Heilongjiang, Harbin	2013-vi-1	XWL	*Eulecanium kuwanai*	*Rosa davurica*	+	+
E3-191C		+	+	Heilongjiang, Harbin	2013-vi-1	XWL	*Eulecanium kuwanai*	*Rosa davurica*	+	+
E3-191D		+	+	Heilongjiang, Harbin	2013-vi-1	XWL	*Eulecanium kuwanai*	*Rosa davurica*		+
E3-191E		+	+	Heilongjiang, Harbin	2013-vi-1	XWL	*Eulecanium kuwanai*	*Rosa davurica*		+
E3 -197A		+	+	Heilongjiang, Harbin	2013-vi-1	XWL	*Eulecanium kuwanai*	*Ulmus pumila*	+	+
E3 -197B		+		Heilongjiang, Harbin	2013-vi-1	XWL	*Eulecanium kuwanai*	*Ulmus pumila*	+	+
E3 -197C				Heilongjiang, Harbin	2013-vi-1	XWL	*Eulecanium kuwanai*	*Ulmus pumila*	+	+
E3 -197D		+	+	Heilongjiang, Harbin	2013-vi-1	XWL	*Eulecanium kuwanai*	*Ulmus pumila*	+	+
E3 -197G				Heilongjiang, Harbin	2013-vi-1	XWL	*Eulecanium kuwanai*	*Ulmus pumila*	+	+
E3-180A		+	+	Inner Mongolia, Baotou	2013-v-22	LHB,WXB,ZX	*Eulecanium kuwanai*	*Sophora japonica*	+	+
E3-180B		+	+	Inner Mongolia, Baotou	2013-v-22	LHB,WXB,ZX	*Eulecanium kuwanai*	*Sophora japonica*		+
E3-180E		+	+	Inner Mongolia, Baotou	2013-v-22	LHB,WXB,ZX	*Eulecanium kuwanai*	*Sophora japonica*		+
E2-035C		+	+	Inner Mongolia, Hohhot	2012-v-26	HBL	*Euiecanium Kuwanai*	*Sophora japonica*	+	+
E2-035D		+	+	Inner Mongolia, Hohhot	2012-v-26	HBL	*Euiecanium Kuwanai*	*Sophora japonica*	+	+
E2-035E		+	+	Inner Mongolia, Hohhot	2012-v-26	HBL	*Euiecanium Kuwanai*	*Sophora japonica*	+	
E2-035F		+	+	Inner Mongolia, Hohhot	2012-v-26	HBL	*Euiecanium Kuwanai*	*Sophora japonica*	+	
E2-035G		+	+	Inner Mongolia, Hohhot	2012-v-26	HBL	*Euiecanium Kuwanai*	*Sophora japonica*	+	
E2-035H		+	+	Inner Mongolia, Hohhot	2012-v-26	HBL	*Euiecanium Kuwanai*	*Sophora japonica*	+	
T-41		+	+	Inner Mongolia, Hohhot	2012-v-26	HBL	*Euiecanium Kuwanai*	*Sophora japonica*		
NM-E-001		+	+	Inner Mongolia, Hohhot	2012-v-26	HBL	*Eulecanium giganteum*	*Sophora japonica*		
NM-E-002		+	+	Inner Mongolia, Hohhot	2012-v-26	HBL	*Eulecanium giganteum*	*Sophora japonica*		
NM-E-003		+	+	Inner Mongolia, Hohhot	2012-v-26	HBL	*Eulecanium giganteum*	*Sophora japonica*		
11-023		+	+	Shanxi, Taiyuan	2007-v-19	JL	*Eulecanium kuwanai*	*Sophora japonica*	+	+
SXE1	SXE1			Shanxi, Taiyuan	2007-v-13	JL	*Eulecanium kuwanai*	*Sophora japonica*	+	+
SXE2	SXE2			Shanxi, Taiyuan	2007-v-13	JL	*Eulecanium kuwanai*	*Sophora japonica*		+
SXE3	SXE3			Shanxi, Taiyuan	2007-v-13	JL	*Eulecanium kuwanai*	*Sophora japonica*	+	+
SXE4	SXE4			Shanxi, Taiyuan	2007-v-13	JL	*Eulecanium kuwanai*	*Sophora japonica*	+	+
SXE5	SXE5	+	+	Shanxi, Taiyuan	2007-v-1	JL	*Eulecanium kuwanai*	*Sophora japonica*	+	
SXE6	SXE6	+	+	Shanxi, Taiyuan	2007-v-1	JL	*Eulecanium kuwanai*	*Sophora japonica*	+	
SXE7	SXE7	+	+	Shanxi, Taiyuan	2007-v-1	JL	*Eulecanium kuwanai*	*Sophora japonica*	+	
SXE8	SXE8	+	+	Shanxi, Taiyuan	2007-v-1	JL	*Eulecanium kuwanai*	*Sophora japonica*	+	
SXE9	SXE9	+		Shanxi, Taiyuan	2007-v-1	JL	*Eulecanium kuwanai*	*Sophora japonica*	+	
SXE10	SXE10	+	+	Shanxi, Taiyuan	2007-v-1	JL	*Eulecanium kuwanai*	*Sophora japonica*	+	
SXR1	SXR1	+	+	Shanxi, Linfen	2007-v-17	JL	*Eulecanium kuwanai*	*Sophora japonica*	+	+
SXR2	SXR2	+	+	Shanxi, Linfen	2007-v-17	JL	*Eulecanium kuwanai*	*Sophora japonica*	+	
HNE1	HNE1		+	Henan: Zhengzhou,	2007-v-4	XW	*Euiecanium Kuwanai*	*Sophora japonica*	+	+
HNE2	HNE2		+	Henan: Zhengzhou,	2007-v-4	XW	*Euiecanium Kuwanai*	*Sophora japonica*	+	+
HNE4	HNE4	+	+	Henan: Zhengzhou,	2007-v-4	XW	*Eulecanium kuwanai*	*Sophora japonica*	+	+
HNE5	HNE5	+	+	Henan: Zhengzhou,	2007-v-4	XW	*Eulecanium kuwanai*	*Sophora japonica*	+	+
HNE6				Henan: Zhengzhou,	2007-v-4	XW	*Eulecanium kuwanai*	*Sophora japonica*		+
HNE7				Henan: Zhengzhou,	2007-v-4	XW	*Eulecanium kuwanai*	*Sophora japonica*		+
SDE1	SDE1		+	Shandong, Taian	2008-v-11	YZZ	*Euiecanium Kuwanai*	*Sophora japonica*	+	+
SDE3	SDE3		+	Shandong, Taian	2008-v-11	YZZ	*Euiecanium Kuwanai*	*Sophora japonica*	+	+
SDE7	SDE7	+	+	Shandong: Taian	2008-v-11	YZZ	*Eulecanium kuwanai*	*Sophora japonica*	+	+

SDE8	SDE8	+	+	Shandong, Taian	2008-v-11	YZZ	*Euiecanium Kuwanai*	*Sophora japonica*	+	+
SDEG				Shandong, Taian	2008-v-10	YZZ	*Euiecanium Kuwanai*	*Albizzia julibrissn*	+	
SDEG1	SDEG1		+	Shandong, Taian	2008-v-10	YZZ	*Eulecanium giganteum*	*Albizzia julibrissn*	+	+
SDEG2	SDEG2		+	Shandong, Taian	2008-v-10	YZZ	*Eulecanium giganteum*	*Albizzia julibrissn*	+	+
SDEG4	SDEG4	+	+	Shandong, Taian	2008-v-10	YZZ	*Eulecanium giganteum*	*Albizzia julibrissn*	+	
SDEG5	SDEG5	+	+	Shandong: Taian	2008-v-11	YZZ	*Eulecanium kuwanai*	*Albizzia julibrissn*	+	
E3-254		+	+	Beijing, Haidian	2012-v-9	YW	*Eulecanium kuwanai*	*Ulmus pumila*		
E3-255		+	+	Beijing, Haidian	2012-v-9	YW	*Eulecanium kuwanai*	*Ulmus pumila*		
E3-257		+	+	Beijing, Haidian	2012-v-9	YW	*Eulecanium kuwanai*	*Ulmus pumila*		
E3-257A		+		Beijing, Haidian	2012-v-9	YW	*Eulecanium kuwanai*	*Ulmus pumila*		
E3-254A		+		Beijing, Xiangshan	2013-v-11	WY, ZLL, WXE	*Eulecanium kuwanai*	*Juglans* sp.		
E3-255A		+		Beijing, Xiangshan	2013-v-11	WY, ZLL, WXE	*Eulecanium kuwanai*	*Juglans* sp.		
08-93A	BJ0893A	+	+	Beijing, Xiangshan	2010-vi-1	YZZ	*Eulecanium kuwanai*	*Sophora japonica*	+	
08-93B	BJ0893B	+		Beijing, Xiangshan	2010-vi-1	YZZ	*Eulecanium kuwanai*	*Sophora japonica*	+	
BJE2	BJE2		+	Beijing, Haidian	2008-vi-1	YZZ	*Eulecanium kuwanai*	*Ulmus pumila*	+	
E4-306A		+	+	Beijing, Shijingshan	2014-v-15	YW	*Eulecanium kuwanai*	*Ulmus pumila*	+	
E4-306B				Beijing, Shijingshan	2014-v-15	YW	*Eulecanium kuwanai*	*Ulmus pumila*	+	
E2-062 A		+	+	Heilongjiang, Harbin	2011-vi-6	XWL	*Rhodococcus sariuoni*	*Prunus tomentosa*	+	+
E2-062 C		+	+	Heilongjiang, Harbin	2011-vi-6	XWL	*Rhodococcus sariuoni*	*Prunus tomentosa*	+	+
E2-062 D		+	+	Heilongjiang, Harbin	2011-vi-6	XWL	*Rhodococcus sariuoni*	*Prunus tomentosa*	+	+
E2-050		+	+	Heilongjiang, Harbin	2012-vi-6	XWL	*Rhodococcus sariuoni*	*Prunus tomentosa*		
E2-050 B		+	+	Heilongjiang, Harbin	2011-vi-9	XWL	*Rhodococcus sariuoni*	*Prunus tomentosa*	+	+
E2-050 C		+		Heilongjiang, Harbin	2011-vi-9	XWL	*Rhodococcus sariuoni*	*Prunus tomentosa*	+	+
E2-064 A		+	+	Heilongjiang, Harbin	2012-vi-6	XWL	*Rhodococcus sariuoni*	*Cerasus tomentosa*	+	+
E2-065 A		+	+	Heilongjiang, Harbin	2012-vi-6	XWL	*Rhodococcus sariuoni*	*Cerasus tomentosa*	+	+
E2-065 B				Heilongjiang, Harbin	2012-vi-6	XWL	*Rhodococcus sariuoni*	*Cerasus tomentosa*		+
E2-065 C		+	+	Heilongjiang, Harbin	2012-vi-6	XWL	*Rhodococcus sariuoni*	*Cerasus tomentosa*	+	+
HLJ-R001		+		Heilongjiang, Harbin	2012-vi-6	XWL	*Rhodococcus sariuoni*	*Amygdalus triloba*		
HLJ-R002		+	+	Heilongjiang, Harbin	2012-vi-6	XWL	*Rhodococcus sariuoni*	*Amygdalus triloba*		
HLJ-R003		+	+	Heilongjiang, Harbin	2012-vi-6	XWL	*Rhodococcus sariuoni*	*Amygdalus triloba*		
HLJ-R004		+	+	Heilongjiang, Harbin	2012-vi-6	XWL	*Rhodococcus sariuoni*	*Amygdalus triloba*		
E3-295		+	+	Heilongjiang, Suhua	2014-v-28	YW	*Rhodococcus sariuoni*	*Padus racemosa*	+	
0820A	HJ820A	+	+	Heilongjiang, Harbin	2007-vi-15	YZZ	*Rhodococcus sariuoni*	*Prunus persica*	+	+
0820B	HJ820B	+	+	Heilongjiang, Harbin	2007-vi-15	YZZ	*Rhodococcus sariuoni*	*Prunus persica*	+	
0820C	HJ820C	+	+	Heilongjiang, Harbin	2007-vi-15	YZZ	*Rhodococcus sariuoni*	*Prunus persica*	+	
E3-198A		+	+	HeilongJiang, Harbin	2012-vi-6	XWL	*Rhodococcus sariuoni*	*Prunus cerasifera*	+	+
E3-198B		+	+	HeilongJiang, Harbin	2012-vi-6	XWL	*Rhodococcus sariuoni*	*Prunus cerasifera*	+	+
E3-198C		+	+	HeilongJiang, Harbin	2012-vi-6	XWL	*Rhodococcus sariuoni*	*Prunus cerasifera*	+	+
E3-198D		+	+	HeilongJiang, Harbin	2012-vi-6	XWL	*Rhodococcus sariuoni*	*Prunus cerasifera*	+	+
11-010		+	+	Jilin, Changchun	2011-vi-9	YW	*Rhodococcus sariuoni*	*Prunus persica*	+	+
11-010B	JL10B	+	+	Jilin, Changchun	2011-vi-9	YW	*Rhodococcus sariuoni*	*Prunus persica*	+	
11-010C	JL10C	+	+	Jilin, Changchun	2011-vi-9	YW	*Rhodococcus sariuoni*	*Prunus persica*	+	
11-010D	JL10D	+	+	Jilin, Changchun	2011-vi-9	YW	*Rhodococcus sariuoni*	*Prunus persica*	+	
11-010E	JL10E	+	+	Jilin, Changchun	2011-vi-9	YW	*Rhodococcus sariuoni*	*Prunus persica*	+	
11-009A	SD09A	+	+	Shandong, Linyi	2011-v-9	XMY	*Rhodococcus sariuoni*	*Crataegus pinnatifida*	+	+
11-009B	SD09B		+	Shandong, Linyi	2011-v-9	XMY	*Rhodococcus sariuoni*	*Crataegus pinnatifida*	+	
11-009C	SD09C		+	Shandong, Linyi	2011-v-9	XMY	*Rhodococcus sariuoni*	*Crataegus pinnatifida*	+	
11-022		+		Shandong, Linyi	2011-v-9	XMY	*Rhodococcus sariuoni*	*Crataegus pinnatifida*	+	+
SD-RA-001		+	+	Shandong, Linyi	2011-iv-30	XMY	*Rhodococcus sariuoni*	*Crataegus pinnatifida*		
SD-RA-002		+	+	Shandong, Linyi	2011-iv-30	XMY	*Rhodococcus sariuoni*	*Crataegus pinnatifida*		
SD-RA-003		+		Shandong, Linyi	2011-iv-30	XMY	*Rhodococcus sariuoni*	*Crataegus pinnatifida*		
E3-216		+	+	shandong, taishan,	2010-v-16	DJ, ZYZ	*Rhodococcus sariuoni*	*Prunus cerasifera*		
E3-217		+	+	shandong, taishan,	2010-v-16	DJ, ZYZ	*Rhodococcus sariuoni*	*Prunus cerasifera*		
E3-218		+	+	shandong, taishan,	2010-v-16	DJ, ZYZ	*Rhodococcus sariuoni*	*Prunus cerasifera*		
E3-219		+	+	shandong, taishan,	2010-v-16	DJ, ZYZ	*Rhodococcus sariuoni*	*Prunus cerasifera*		
11-020		+	+	shandong, taishan,	2010-v-16	DJ, ZYZ	*Rhodococcus sariuoni*	*Prunus cerasifera*	+	+
SDER1	SDER1		+	Shandong, Taian	2008-v-9	YZZ	*Rhodococcus sariuoni*	*Prunus cerasifera*	+	+
SDER2	SDER2		+	Shandong, Taian	2008-v-9	YZZ	*Rhodococcus sariuoni*	*Prunus cerasifera*	+	+
SDER7A				Shandong, Taian	2008-v-9	YZZ	*Rhodococcus sariuoni*	*Prunus cerasifera*	+	+
SDER7B				Shandong, Taian	2008-v-9	YZZ	*Rhodococcus sariuoni*	*Prunus cerasifera*	+	
SDER8A		+		Shandong, Taian	2008-v-9	YZZ	*Rhodococcus sariuoni*	*Prunus cerasifera*	+	+
SDER8B				Shandong, Taian	2008-v-9	YZZ	*Rhodococcus sariuoni*	*Prunus cerasifera*	+	
11-011A	SD011A	+	+	Shandong, Weifang	2011-v-9	YZZ	*Rhodococcus sariuoni*	*Prunus persica*	+	+
11-011B	SD011B	+	+	Shandong, Weifang	2011-v-9	YZZ	*Rhodococcus sariuoni*	*Prunus persica*	+	
11-011C	SD011C	+	+	Shandong, Weifang	2011-v-9	YZZ	*Rhodococcus sariuoni*	*Prunus persica*	+	
11-011D	SD011D	+	+	Shandong, Weifang	2011-v-9	YZZ	*Rhodococcus sariuoni*	*Prunus persica*	+	
11-018		+	+	Shandong, Weifang	2011-v-9	DJ, ZYZ	*Rhodococcus sariuoni*	*Malus pumila*	+	+
11-008A	SH008A	+	+	Shaanxi, Xianyang	2011-v-15	Feng Yuan	*Rhodococcus sariuoni*	*Malus sieversii*	+	+
11-008B	SH008B	+	+	Shaanxi, Xianyang	2011-v-15	Feng Yuan	*Rhodococcus sariuoni*	*Malus sieversii*	+	
E3-185A		+	+	Qinghai, Xining	2013-v-26	LHB,WXB,ZX	*Rhodococcus sariuoni*	*Armeniaca vulgaris*	+	+
E3-185B		+	+	Qinghai, Xining	2013-v-26	LHB,WXB,ZX	*Rhodococcus sariuoni*	*Armeniaca vulgaris*	+	+
E3-190A		+		Qinghai, Xining	2013-v-26	LHB,WXB,ZX	*Rhodococcus sariuoni*	*Prunus cerasifera*	+	
E3-190B				Qinghai, Xining	2013-v-26	LHB,WXB,ZX	*Rhodococcus sariuoni*	*Prunus cerasifera*	+	
0850A	QH850A	+	+	Qinghai, Xining	2007-vi-22	YZZ	*Rhodococcus sariuoni*	*Prunus persica*	+	+
0850B	QH850B			Qinghai, Xining	2013-v-26	LHB,WXB	*Rhodococcus sariuoni*	*Prunus cerasifera*	+	
0850C				Qinghai, Xining	2013-v-26	LHB,WXB	*Rhodococcus sariuoni*	*Prunus cerasifera*	+	
11-046		+	+	Beijing, Haidian	2011-vi-4	YZZ	*Rhodococcus sariuoni*	*Malus sieversii*	+	
11-007A	BJ007A	+	+	Beijing, Haidian	2011-v-15	YZZ	*Rhodococcus sariuoni*	*Malus spectabilis*	+	+
11-007B	BJ007B	+	+	Beijing, Haidian	2011-v-15	YZZ	*Rhodococcus sariuoni*	*Malus spectabilis*	+	
BJER1	BJER1		+	Beijing, Haidian	2006-v-1	YZZ	*Rhodococcus sariuoni*	*Malus spectabilis*	+	+
BJER2	BJER2	+	+	Beijing, Haidian	2011-v-15	YZZ	*Rhodococcus sariuoni*	*Malus spectabilis*	+	
BJER3	BJER3	+	+	Beijing, Haidian	2011-v-15	YZZ	*Rhodococcus sariuoni*	*Malus spectabilis*	+	
BJER4	BJER4	+	+	Beijing, Haidian	2011-v-15	YZZ	*Rhodococcus sariuoni*	*Malus spectabilis*	+	
BJER5	BJER5	+	+	Beijing, Haidian	2011-v-15	YZZ	*Rhodococcus sariuoni*	*Malus spectabilis*	+	
BJEG1	BJEG1		+	Beijing, Haidian	2006-v-1	YZZ	*Rhodococcus sariuoni*	*Sophora japonica*	+	+

BJEG2	BJEG2	+	+	Beijing, Haidian	2006-v-1	YZZ	*Rhodococcus sariuoni*	*Sophora japonica*

Note: (+: extracted information in the study；Old Code: all the sample we list in the Chesters et al., 2012)

Table S2. **Pearson's product-moment correlation coefficients for all measurements of Encyrtus**

	SL	SW	PL	PW	F1L	F1W	F2L	F2W	F3L	F3W	F4L	F4W	F5L	F5W	F6L	F6W	CL	CW	FT	MT	MTS	HT	OV	PVL	SVL
SL	1	0.52	0.67	0.59	0.88	0.45	0.83	0.49	0.8	0.48	0.78	0.46	0.75	0.53	0.61	0.57	0.71	0.57	0.86	0.92	0.79	0.88	0.64	0.83	0.83
SW	0.52	1	0.52	0.72	0.46	0.45	0.56	0.49	0.53	0.46	0.44	0.46	0.46	0.51	0.38	0.57	0.52	0.45	0.57	0.52	0.57	0.53	0.42	0.48	0.46
PL	0.67	0.52	1	0.66	0.64	0.57	0.65	0.56	0.65	0.6	0.53	0.59	0.55	0.63	0.52	0.65	0.58	0.56	0.68	0.68	0.68	0.7	0.44	0.68	0.7
PW	0.59	0.72	0.66	1	0.58	0.68	0.63	0.65	0.63	0.62	0.58	0.63	0.54	0.69	0.54	0.69	0.59	0.5	0.7	0.65	0.68	0.68	0.43	0.63	0.65
F1L	0.88	0.46	0.64	0.58	1	0.5	0.82	0.49	0.76	0.5	0.78	0.45	0.71	0.53	0.61	0.56	0.68	0.55	0.83	0.88	0.78	0.83	0.64	0.81	0.8
F1W	0.45	0.45	0.57	0.68	0.5	1	0.53	0.84	0.53	0.82	0.44	0.77	0.5	0.8	0.49	0.75	0.49	0.63	0.65	0.54	0.6	0.64	0.3	0.61	0.62
F2L	0.83	0.56	0.65	0.63	0.82	0.53	1	0.58	0.87	0.54	0.86	0.55	0.79	0.56	0.73	0.63	0.65	0.51	0.82	0.84	0.79	0.78	0.64	0.74	0.73
F2W	0.49	0.49	0.56	0.65	0.49	0.84	0.58	1	0.54	0.9	0.47	0.85	0.52	0.83	0.51	0.77	0.51	0.63	0.66	0.56	0.6	0.65	0.29	0.63	0.65
F3L	0.8	0.53	0.65	0.63	0.76	0.53	0.87	0.54	1	0.53	0.82	0.51	0.81	0.56	0.71	0.58	0.68	0.5	0.84	0.83	0.76	0.81	0.64	0.74	0.74
F3W	0.48	0.46	0.6	0.62	0.5	0.82	0.54	0.9	0.53	1	0.43	0.86	0.48	0.87	0.5	0.82	0.55	0.69	0.63	0.56	0.61	0.66	0.3	0.63	0.65
F4L	0.78	0.44	0.53	0.58	0.78	0.44	0.86	0.47	0.82	0.43	1	0.43	0.79	0.46	0.68	0.49	0.65	0.4	0.79	0.78	0.72	0.71	0.65	0.66	0.66
F4W	0.46	0.46	0.59	0.63	0.45	0.77	0.55	0.85	0.51	0.86	0.43	1	0.46	0.88	0.5	0.85	0.52	0.62	0.59	0.52	0.57	0.6	0.27	0.59	0.6
F5L	0.75	0.46	0.55	0.54	0.71	0.5	0.79	0.52	0.81	0.48	0.79	0.46	1	0.51	0.8	0.54	0.63	0.47	0.76	0.75	0.68	0.72	0.58	0.68	0.66
F5W	0.53	0.51	0.63	0.69	0.53	0.8	0.56	0.83	0.56	0.87	0.46	0.88	0.51	1	0.49	0.91	0.57	0.68	0.63	0.58	0.61	0.67	0.33	0.65	0.67
F6L	0.61	0.38	0.52	0.54	0.61	0.49	0.73	0.51	0.71	0.5	0.68	0.5	0.8	0.49	1	0.53	0.51	0.47	0.66	0.65	0.64	0.6	0.51	0.59	0.6
F6W	0.57	0.57	0.65	0.69	0.56	0.75	0.63	0.77	0.58	0.82	0.49	0.85	0.54	0.91	0.53	1	0.6	0.67	0.64	0.59	0.64	0.64	0.36	0.64	0.62
CL	0.71	0.52	0.58	0.59	0.68	0.49	0.65	0.51	0.68	0.55	0.65	0.52	0.63	0.57	0.51	0.6	1	0.4	0.71	0.69	0.62	0.71	0.53	0.65	0.62
CW	0.57	0.45	0.56	0.5	0.55	0.63	0.51	0.63	0.5	0.69	0.4	0.62	0.47	0.68	0.47	0.67	0.4	1	0.59	0.62	0.61	0.65	0.37	0.64	0.67
FT	0.86	0.57	0.68	0.7	0.83	0.65	0.82	0.66	0.84	0.63	0.79	0.59	0.76	0.63	0.66	0.64	0.71	0.59	1	0.9	0.85	0.89	0.62	0.85	0.86
MT	0.92	0.52	0.68	0.65	0.88	0.54	0.84	0.56	0.83	0.56	0.78	0.52	0.75	0.58	0.65	0.59	0.69	0.62	0.9	1	0.85	0.94	0.64	0.86	0.87
MTS	0.79	0.57	0.68	0.68	0.78	0.6	0.79	0.6	0.76	0.61	0.72	0.57	0.68	0.61	0.64	0.64	0.62	0.61	0.85	0.85	1	0.81	0.61	0.74	0.76
HT	0.88	0.53	0.7	0.68	0.83	0.64	0.78	0.65	0.81	0.66	0.71	0.6	0.72	0.67	0.6	0.64	0.71	0.65	0.89	0.94	0.81	1	0.58	0.88	0.9
OV	0.64	0.42	0.44	0.43	0.64	0.3	0.64	0.29	0.64	0.3	0.65	0.27	0.58	0.33	0.51	0.36	0.53	0.37	0.62	0.64	0.61	0.58	1	0.54	0.52
PVL	0.83	0.48	0.68	0.63	0.81	0.61	0.74	0.63	0.74	0.63	0.66	0.59	0.68	0.65	0.59	0.64	0.65	0.64	0.85	0.86	0.74	0.88	0.54	1	0.96
SVL	0.83	0.46	0.7	0.65	0.8	0.62	0.73	0.65	0.74	0.65	0.66	0.6	0.66	0.67	0.6	0.62	0.62	0.67	0.86	0.87	0.76	0.9	0.52	0.96	1

Table S3. Shape PCA components

	shape.PC1	shape.PC2	shape.PC3	shape.PC4	shape.PC5	shape.PC6	shape.PC7	shape.PC8	shape.PC9	shape.PC10	shape.PC11	shape.PC12	shape.PC13
Variance	0.034	0.0169	0.0111	0.0079	0.0064	0.0057	0.0054	0.0044	0.0036	0.0033	0.0032	0.0027	0.0022
Proportion of Variance	0.2829	0.1406	0.0923	0.066	0.0534	0.0477	0.0452	0.0369	0.0303	0.0274	0.0266	0.0222	0.0183
Cumulative Proportion	0.2829	0.4235	0.5159	0.5819	0.6353	0.683	0.7282	0.7651	0.7954	0.8228	0.8494	0.8716	0.8899

	shape.PC14	shape.PC15	shape.PC16	shape.PC17	shape.PC18	shape.PC19	shape.PC20	shape.PC21	shape.PC22	shape.PC23	shape.PC24	shape.PC25
Variance	0.0022	0.0019	0.0017	0.0015	0.0014	0.0012	0.0011	0.0009	0.0006	0.0004	4.00E-04	0
Proportion of Variance	0.018	0.0159	0.014	0.0121	0.0113	0.0098	0.0094	0.0075	0.0054	0.0036	3.00E-03	0
Cumulative Proportion	0.9079	0.9238	0.9379	0.9499	0.9613	0.9711	0.9805	0.988	0.9934	0.997	1.00E+00	1

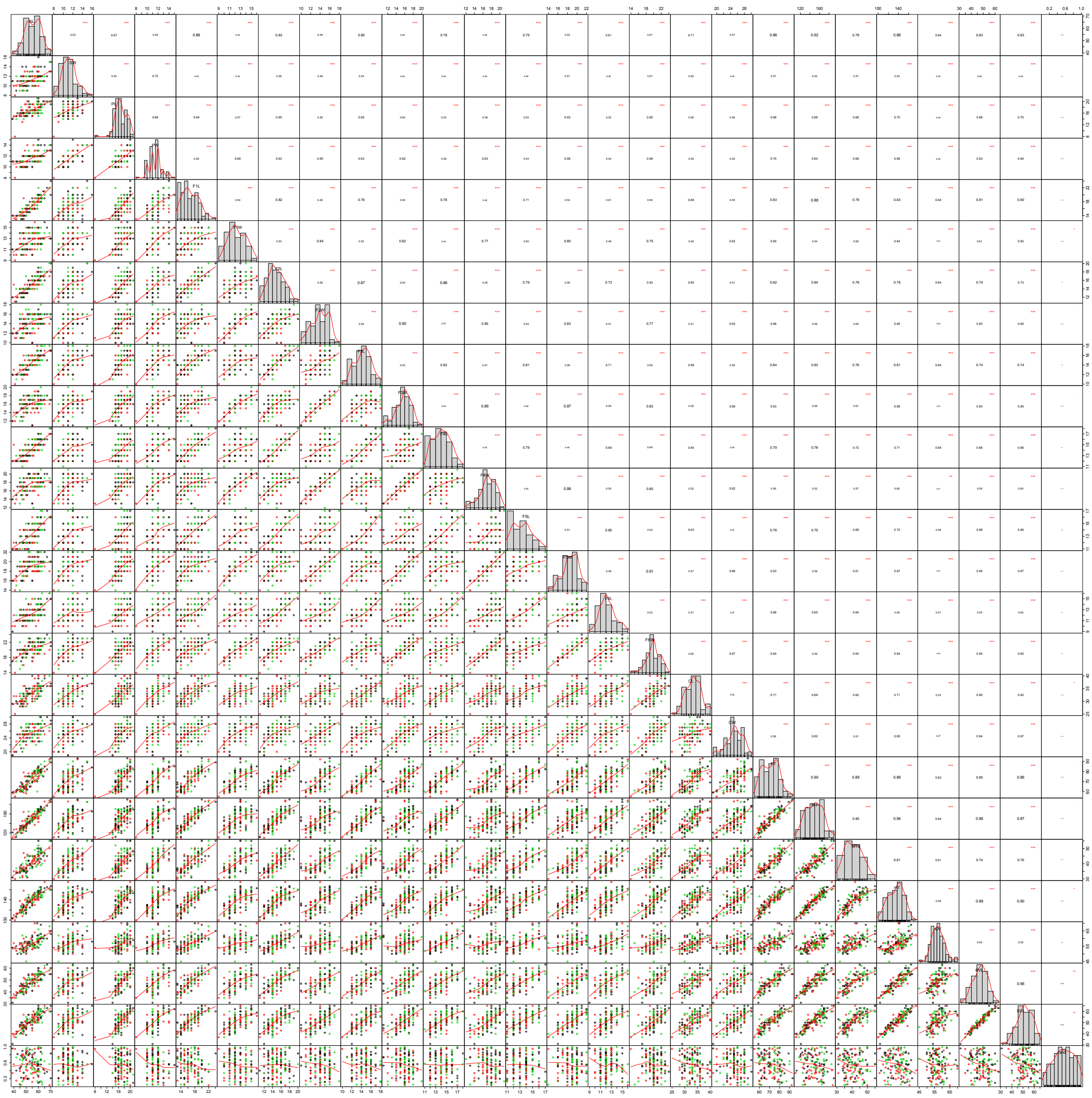

Figure S1. Matrix scatterplots and Pearson product-moment correlation coefficients of *Encyrtus* spp. based on 25 of the morphological characters.

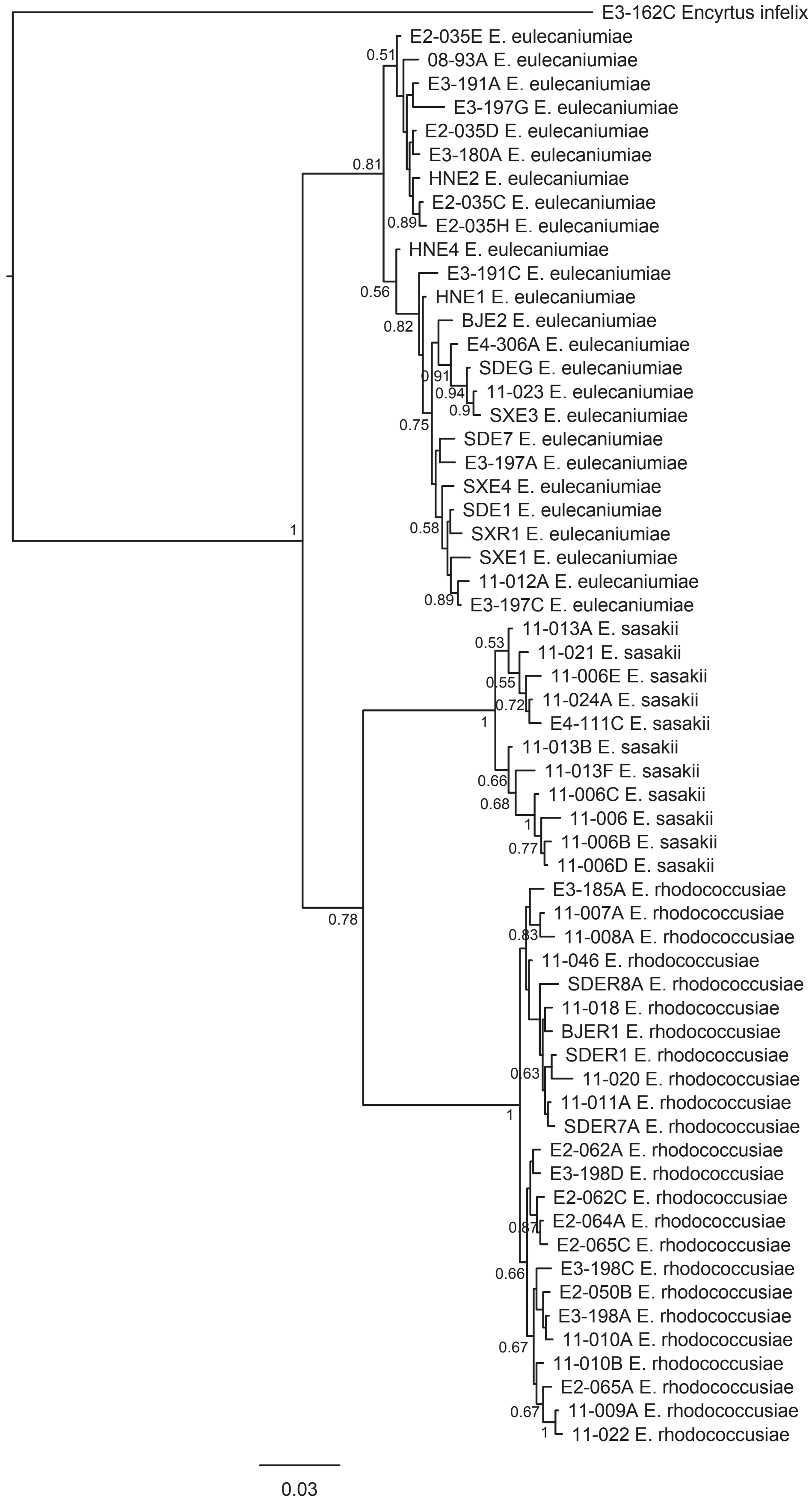

Figure S2. Bayesian inference phylogeny of *Encyrtus* spp. constructed by Mrbayes based on COI dataset.

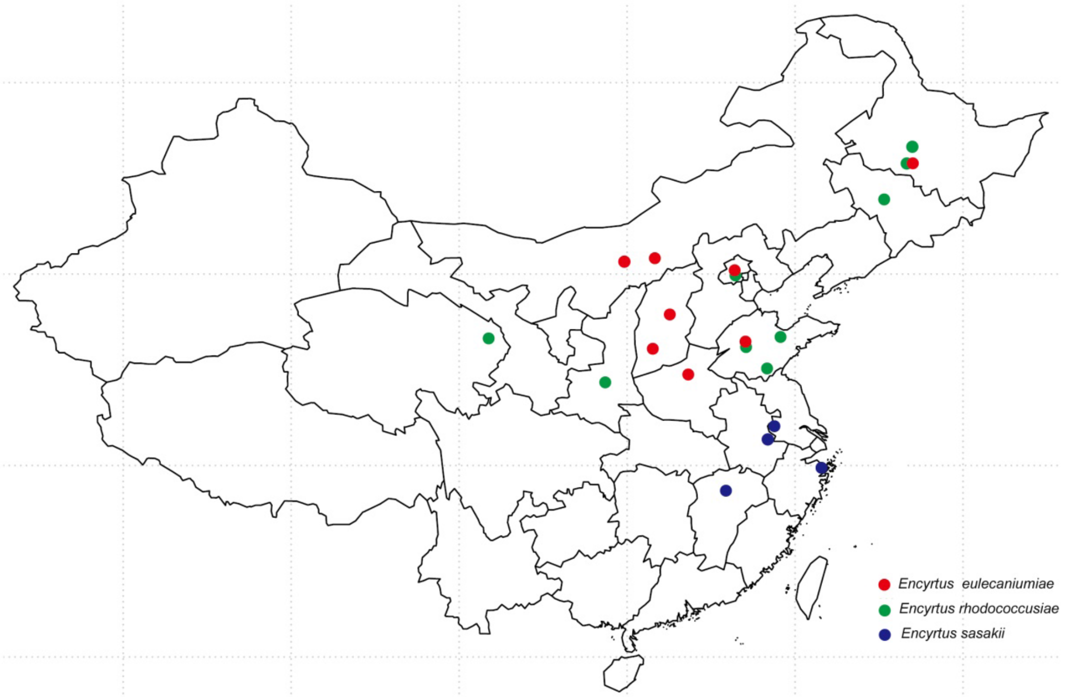

Figure S3. Sampling sites of *Encyrtus* spp. in China. This figure was drawn in "R" using the mapdata package created by R Development Core Team[67], and the map will not have a copyright dispute.
